# Shengmai Injection Suppresses Angiotensin II-Induced Cardiomyocyte Hypertrophy and Apoptosis *via* Activation of the AMPK Signaling Pathway Through Energy-Dependent Mechanisms

**DOI:** 10.3389/fphar.2019.01095

**Published:** 2019-09-20

**Authors:** Yiping Li, Xiaofen Ruan, Xiaowen Xu, Cha Li, Tingting Qiang, Hua Zhou, Junjie Gao, Xiaolong Wang

**Affiliations:** ^1^Cardiovascular Department of Traditional Chinese Medicine, Shuguang Hospital Affiliated to Shanghai University of Traditional Chinese Medicine, Shanghai, China; ^2^Cardiovascular Research Institute of Traditional Chinese Medicine, Shuguang Hospital Affiliated to Shanghai University of Traditional Chinese Medicine, Shanghai, China

**Keywords:** Shengmai injection, cardiomyocyte hypertrophy, apoptosis, energy metabolism, AMPK signaling pathway

## Abstract

Shengmai injection (SMI), a traditional Chinese herbal medicine extracted from Panax ginseng C.A. Mey., *Ophiopogon japonicus * (Thunb.) Ker Gawl., and *Schisandra chinensis* (Turcz.) Baill., has been used to treat acute and chronic heart failure. This study aimed to further clarify the effects of SMI on energy metabolism. SMI could improve cell-survival rate and also reduce myocardial cell hypertrophy and apoptosis. Mitochondria are important sites of cellular energy metabolism, and SMI protects mitochondrial function which was evaluated by mitochondrial ultrastructure, mitochondrial respiratory control ratio (RCR), and mitochondrial membrane potential (ΔΨm) in this study. The expression levels of adenosine triphosphate (ATP), adenosine diphosphate (ADP), and phosphocreatine (PCr) increased. The expression levels of free fatty acid oxidation [carnitine palmitoyltransferase-1 (CPT-1)], glucose oxidation [glucose transporter-4 (GLUT-4)], and mitochondrial biogenesis-related genes (peroxisome proliferator-activated receptor-γ coactivator-1α [PGC-1α]) were upregulated after SMI treatment. AMP-activated protein kinase (AMPK) is an important signaling pathway regulating energy metabolism and also can regulate the above-mentioned indicators. In the present study, SMI was found to promote phosphorylation of AMPK. However, the effects of SMI on fatty acid, glucose oxidation, mitochondrial biogenesis, as well as inhibiting apoptosis of hypertrophic cardiomyocytes were partly blocked by AMPK inhibitor–compound C. Moreover, decreased myocardial hypertrophy and apoptosis treated by SMI were inhibited by AMPK knockdown with shAMPK to a certain degree and AMPK knockdown almost abolished the SMI-induced increase in the expression of GLUT-4, CPT-1, and PGC-1α. These data suggest that SMI suppressed Ang II–induced cardiomyocyte hypertrophy and apoptosis via activation of the AMPK signaling pathway through energy-dependent mechanisms.

## Introduction

Heart failure (HF) is a major public health concern, affecting more than 6.2 million people in the USA (Benjamin et al., 2019) and more than 23 million people worldwide (Go et al., 2013). The pathogenesis of HF is pathological cardiac remodeling, manifested as left ventricular dilatation, myocardial cell hypertrophy, and interstitial fibrosis (Opie et al., 2006; Shah et al., 2012). Among them, cardiomyocyte hypertrophy is the main cause of decreased myocardial cell contractility and decreased blood supply capacity (Roman et al., 1997). A previous study confirmed the activation of the neuroendocrine system, especially the renin–angiotensin–aldosterone system (RAAS), which is a key mechanism leading to cardiomyocyte hypertrophy. Cutting off RAAS to reduce cardiomyocyte hypertrophy has become the basis for preventing and treating of HF (Tamura et al., 2000). Classical HF treatments, in particular angiotensin-converting enzyme indicators/angiotensin receptor blockers and beta-blockers, have been successfully conducted. However, the 1-year mortality rate of HF has only slightly declined, and its 5-year mortality rate has not declined during the last 10 years (Benjamin et al., 2019).

Alterations in cardiac energy metabolism contribute to the severity of HF and cardiac hypertrophy. Myocardial oxygen consumption is very high in HF and cardiac hypertrophy, exhibiting an energy deficit (containing 30–40% less ATP compared with a healthy heart) due to altered energy substrate availability and impaired mitochondrial oxidative metabolism (De Jong and Lopaschuk, 2017). At the same time, important factors are involved in fatty acid metabolism and ATP synthesis, such as carnitine palmitoyl transferase 1 (CPT-1), peroxisome proliferator–activated receptorγ coactivator1α (PGC-1α), and malonyl-CoA decarboxylase, which were abnormally expressed during cardiac hypertrophy (Haynes and Campbell, 2014; Torrens et al., 2017). Among them, CPT-1 controls the rate-limiting step of mitochondrial fatty acid oxidation, and PGC-1α controls mitochondrial biosynthesis and the entire pathway of ATP synthesis (Hoppel et al., 2009). In addition to fatty acid oxidation, glycolysis is an alternate source of ATP production. Glucose is taken up by cardiomyocytes *via* glucose transporter1 (GLUT-1) and glucose transporter4 (GLUT-4); GLUT-4 is primarily responsible for the insulin-dependent uptake of glucose (Fan et al., 2011; Dolinsky et al., 2016; De Jong and Lopaschuk, 2017). After knocking out the glucose carrier GLUT-4 in mice, the heart gradually develops into a hypertrophic phenotype (Luo et al., 2018). Consequently, metabolic agents may be a complementary effective strategy for treating HF (Rosano and Vitale, 2018).

Shengmai is a traditional Chinese herbal medicine. It is a mixture extracted from three herbs: *Panax ginseng* C.A. Mey., *Ophiopogon japonicus* (Thunb.) Ker Gawl., and *Schisandra chinensis* (Turcz.) Baill. It is the most important rescue agent for patients before wide prescription of Western medicine in China. Nowadays, Shengmai is typically produced as a patented drug on the basis of a standardized formula in different forms, including capsule, powder, oral liquid, and injection. It is also typically prescribed in China as a complementary treatment compared with Western medicine recommended for acute HF and chronic HF. Although the poor quality of trials compromised the reliability of the evidence, the efficacy of Shengmai against HF has been demonstrated in several studies (Zhou et al., 2014). A recent clinical trial further confirmed the efficacy that the patients with CHF and coronary artery disease (CAD) could benefit from the integrative treatments with standard medicines plus Shengmai injection (SMI) with respect to the New York Heart Association (NYHA) functional classification, 6-min walking distance (6MWD), 36-item short-form health survey (SF-36) (SF-36) score, and traditional Chinese medicine (TCM) syndrome score (Xian et al., 2016). Previous basic studies have proved that SMI protected myocardial ischemia-reperfusion injury and doxorubicin-induced cardiomyopathy related to improving myocardial energy metabolism (Quaini et al., 2015; Zhan et al., 2015). However, whether it can improve the cardiac function *via* improving the mitochondrial energy metabolism of cardiac hypertrophy remains elusive.

Cardiac hypertrophy is characterized by increased cardiomyocyte size, a higher degree of sarcomere organization, and enhanced gene transcription and translation levels, all of which are closely associated with energy metabolism (Tham et al., 2015). This study established the model of cardiomyocyte hypertrophy induced by Angiotensin (Ang) II to investigate the protective effects of SMI on myocardial energy metabolism and the underlying mechanism, so as to provide a new complementary target for the energy metabolism of HF.

## Materials and Methods

### Chemicals and Treatments

SMI (10 ml/tablet, H17050402; Shanghai Hutchison Pharmaceuticals, Shanghai, China), Ang II (Sigma–Aldrich, St. Louis, MO, USA), and the AMP-activated protein kinase (AMPK) inhibitor compound C (S7306, Selleck Chemicals, Houston, TX, USA) were purchased. All animals used in this study were 1- to 4-day old Sprague–Dawley (SD) rats (Experimental Animal Center, Shanghai University of Traditional Chinese Medicine, Shanghai, China).

Cardiomyocytes were randomly assigned into three groups: blank, Ang II (0.8 µM), and SMI groups (Ang II 0.8 µM + SMI 1/4,000). The cardiomyocytes were pretreated with phosphate-buffered saline (PBS) or SMI for 1 h and then stimulated with 0.8 µM Ang II for 48 h.

To prove that SMI regulates glycolipid metabolism and mitochondrial synthesis through the AMPK pathway, we later divided the cardiomyocytes into five groups: blank, Ang II (0.8 µM), compound C (Ang II 0.8 µM + compound C 0.1uM), SMI (Ang II 0.8 µM + SMI 1/4,000), and SMI + compound C (Ang II 0.8 µM + SMI 1/4,000 + compound C 0.1 µM). Also, the levels of proteins related to energy metabolism were examined, besides flow cytometry analysis of cell apoptosis and apoptosis-related proteins.

### Preparation and Quality Control Standard of SMI

The quality control standard of SMI conformed to the National Drug Standard of the Ministry of Health of China, which specifies that the total amount of ginsenoside Rg1 and ginsenoside Re should not be lower than 0.08 and 0.04mg in a 1 ml injection respectively, as analyzed by high-performance liquid chromatography (HPLC).

To guide the rational use of SMI in clinic, an increasing number of studies focused on the fingerprint analysis of SMI and showed that more than 10 components were determined, including ginsenoside Rg1, ginsenoside Re, ginsenoside Rf, ginsenoside Rb1, ginsenoside Rc, ginsenoside Rh1, ginsenoside Rd, schisandrin, ginsenoside Rg5 (Rk1), and ginsenoside Rh3 (**Supplementary Figures 2–3** and **Supplementary Tables 1–2**).

### Isolation of Neonatal Cardiomyocyte and Cell Models for Hypertrophy

Neonatal rat ventricular myocytes were isolated and cultured from the ventricles of postnatal days 1–3 SD rats. The hearts were removed from mice aseptically, and large vessels and atria were discarded. The ventricles were washed, cut into small pieces in Dulbecco’s modified Eagle’s medium (DMEM), and then digested in 0.25% trypsin in a CO_2_ incubator at 37°C for 15 min. To enrich cardiomyocytes and deplete nonmyocytes, the cells were centrifuged and cultured in DMEM for 2 h, allowing the attachment of nonmyocytes. After the purification process, the cardiomyocytes were resuspended and cultured in DMEM supplemented with 15% fetal bovine serum. The culture medium was renewed after 48 h, and the cardiomyocytes were further cultured for 24 h. Next, the culture medium was changed with serum-free DMEM, and the cardiomyocytes were pretreated with PBS, SMI, or compound C (both diluted with PBS) for 1 h and then stimulated with Ang II (0.8 µM) for 48 h.

### Quantitative Reverse Transcription Polymerase Chain Reaction (RT-qPCR)

Quantitative analysis of mRNA expression of atrial natriuretic peptide (ANP), brain natriuretic peptide (BNP), and β-myosin heavy chain (β-MHC) in cardiomyocytes and cardiac tissues was conducted using RT-qPCR. Total RNA was extracted from cardiomyocytes by using TRIzol reagent (15596-026; Invitrogen, Carlsbad, CA, USA) according to the manufacturer’s protocols. Next, 0.5 µg of total RNA was reversely transcribed using the SuperScript III Reverse Transcriptase (R250-01; Invitrogen, Carlsbad, CA, USA) to obtain cDNA. The SYBR Green PCR Master Mix Kit (CS7561; Invitrogen, Carlsbad, CA, USA) was used to quantify the RNA levels of the hypertrophic markers, such as ANP, BNP, and β-MHC in cardiomyocytes, with glyceraldehyde-3-phosphate dehydrogenase (GAPDH) as an internal control. The RT-qPCR was performed by CFX96TM Real-Time System (Bio-Rad Laboratories, CA, USA) for 40 cycles. The sequences of primers used for amplification were as follows: ANP, 5’-CTCCGATAGATCTGCCCTCTTGAA-3’ and 5’-GGTACCGGAA GCTGTTGCAGCCTA-3’; BNP, 5’-TGATTCTGCTCCTGCT TTTC-3’ and 5’-GTGGATTGTTCTGGAGACTG-3’; β-MHC, 5’-CAGCAGCCCAGTACCTCCGA-3’ and 5’-TGTCATCAGG CACGAAGCAC-3’; GLUT-4, 5’-GCCGGGACACTATACCC TATTC-3’ and 5’-AAGGACCAGTGTCCCAGTCA-3’; CPT-1, 5’-GCAGCTCGCACATTACAAGG-3’ and 5’-CGTTGACTT GGGGTCCATCA-3’; PGC-1α, 5’-CATTCAGGAGCTGGATG GCT-3’ and 5’-TATGTTCGCGGGCTCATTGT-3’; and GAP DH,5’-AAGAATGGTGAAGCAGGC-3’ and 5’-TCCACCAC CAGTTGCTGTA-3.’ Data were analyzed using 2ΔΔCt method for relative quantification. In this study, ΔCt was calculated as Ct (detected mRNA) − Ct (GAPDH), and ΔΔCt was calculated as ΔCt (drug treated) − ΔCt (control), where the control is the group treated with non-drugs. The relative value was obtained by 2-ΔΔCt.

### Cell Viability Assay

The CellTiter-Glo Luminescent Cell Viability Assay Kit (C0068M; Beyotime Institute of Biotechnology, Shanghai, China) was used to test the cell viability according to manufacturer’s protocol. Briefly, the cells were seeded on 96-well plates at 1 × 10^5^ cells/well overnight. The reagent was reconstituted and added to the cells. After mixing on an orbital shaker for 2 min to lyse the cells, the plate was incubated at room temperature for 10 min, and then the luminescent signals were recorded.

### Determination of Cardiomyocyte Area

The cells with a satisfactory growth rate were digested and re-suspended the previous day. They were inoculated in a six-well plate and treated in different groups for 48 h after the liquid exchange. Then, 1 ml of carboxyfluorescein diacetate succinimidyl ester (CFDA-SE) (C0051, Beyotime Institute of Biotechnology, Shanghai, China) was added; stock solution (2×) was added for 10 min at 37°C. The cells were washed one to two times with 1× PBS. After adding 1–2 ml of complete cell culture medium and incubating for 5 min at 37°C, the cells washed one to two times again with 1× PBS. Further 4% paraformaldehyde (1 ml) was added to fix for 30 min, and the cells were washed with 2× PBS two to three times. The cell nuclei were stained with Hoechst 33342 (C1022; Beyotime Institute of Biotechnology, Shanghai, China) solution (final concentration of 1 µg/ml) at room temperature for 5–10 min and washed two to three times with 1× PBS. Next, 1 ml of 1× PBS was added to each well, and the cells were observed and photographed under a fluorescence microscope; Image-Pro Plus software was used to calculate the total area and number of cardiomyocytes in the same field of view and the number of cardiomyocytes in the same field of view.

Average cell area in the same field of view = (total area of the same field of view)/(the number of core cells in the same field of view).

### Detecting Protein Content of Cardiomyocytes by Bicinchoninic Acid Assay

Depending on the number of samples, 50 parts of bicinchoninic acid (BCA) reagent A plus 1 volume of BCA reagent (50:1, P0012, Beyotime Institute of Biotechnology) were mixed to configure the appropriate amount of BCA working solution. Then, 10 µl of protein standard diluted with 100 µl of PBS to a final concentration of 0.5 mg/ml. The standard was added to a 96-well plate and diluted to 20 µl. The sample to be tested was added to the 96-well plate, and the standard was diluted to 20 µl. Further, 200 µl of BCA working solution was added to each well and placed at 37°C for 30 min. The optical density (OD) value at 562 nm. The protein concentration was calculated from the standard curve.

### Bromodeoxyuridine (BrdU) Incorporation

The cells were incubated with culture media containing 10µM 5-bromo-20-deoxybromouridine (BrdU) (Cat. No. B23151, Invitrogen, USA), for 6 h at 37°C; fixed with 3.7% formaldehyde for 15 min; and treated according to the BrdU experimental protocol. Briefly, the medium was removed, and the cells were washed with PBS. The PBS was replaced with 1 ml of Triton X-100 permeabilization buffer for 20 min at room temperature. The permeabilization buffer was removed, and 1 ml of 1N HCl was added, which was incubated for 10 min on ice. The cells were then incubated with 2N HCl for 10 min at room temperature. The acid solution was removed, and the cells were washed three times with Triton X-100 permeabilization buffer. The buffer was then removed and replaced with the staining solution. After an incubation period of 24 h, the anti-BrdU primary antibody (Cat. No. B35130, Invitrogen, USA) was removed, and the cells were washed three times with Triton X-100 permeabilization buffer. Each well was incubated with fluorescently labeled secondary antibody (Cat. No. A-11001, Invitrogen, USA) for 1 h at room temperature. Finally, the cells were imaged with appropriate filters.

### Mitochondria Extraction and Determination of Oxidative Respiratory Function

Genmed medium (2.5 ml) was added into the reaction glass tank, mixed, and sealed. Further, mitochondria, state IV substrate solution, and state III substrate solution were added in a proper sequence following the manufacturer’s protocol. Mitochondrial state III and state IV respiration rates in the closed reaction system were determined using dissolved oxygen electrolytic solution (Orion 4-tar; Thermo Fisher Scientific, MA, USA). The mitochondrial respiratory control ratio (RCR) was calculated according to the following formula: RCR, state III respiration rate/state IV respiration rate.

### Transmission Electron Microscopy

The adherent cells were directly scraped off, and the cell suspension was centrifuged (1,000 rpm, 5 min). The supernatant was discarded, washed twice with PBS, and pelleted by centrifugation. Further, 2.5% glutaraldehyde was added for fixation at 4°C overnight. After washing with 0.1M PBS three times, the supernatant was incubated with 1% citric acid (Pelco, CA, USA) at 4°C for 3 h, washed with buffer three times, and dehydrated with ethanol step by step, followed by the replacement of propylene oxide. The Spurr resin (SPI-Chem, Structure Probe, Inc., PA, USA) was impregnated and embedded. Finally, it was polymerized in an oven at 70°C. The embedded blocks of different materials were placed on an ultrathin slicer (EM UC6; Leica, Wetzlar, Germany) for sectioning. The ultrathin section was 70 nm thick; it was stained with uranyl acetate (SPI-Chem, Structure Probe, Inc. A) and lead citrate (SPI-Chem, Structure Probe, Inc.) and then observed under a transmission electron microscope (JEM1230; JEOL Ltd., Tokyo, Japan).

### JC-1 Stain for Mitochondrial Membrane Potential (㽪Δψm)

Neonatal mouse ventricular myocytes in three groups were cultured in 24-well plates and treated as mentioned earlier. The cells were washed with PBS once, and 0.25 ml of fresh medium was added into each well. JC-1 dye (0.25 ml, C2006; Beyotime Institute of Biotechnology) was added to each well and mixed well. The cells were then incubated in an incubator at 37°C for 20 min. At the end of incubation, the supernatant was discarded, and the cells were washed. The cell culture medium (0.5 ml) was added, and the cells were observed under a fluorescence microscope. Integrated OD (IOD), as calculated by multiplying the area and average density of the fluorescence, was evaluated using Image-Pro Plus 7 software (Media Cybernetics Inc., MD, USA).

### Detection of ATP, ADP, AMP, and PCr Levels in Cardiomyocytes by HPLC Assay

The supernatant (400 µl) was collected from different groups and centrifuged again at 4,000 rpm for 10 min at 4°C; the supernatant was filtered with a 0.45-µm membrane. The prepared supernatant was loaded onto a 10-A HPLC detector (Monolithic RP-C18, 2.0 × 50 mm^2^; Merck, Darmstadt, Germany with Waters UPLC Q-trap5500 [1024985-AX; AB SCIEX, MA, USA]), followed by separation and detection at a wavelength of 254 nm. The levels of adenosine triphosphate (ATP) (99%, Sigma–Aldrich, MO, USA), adenosine diphosphate (ADP) (99%, Sigma), adenosine monophosphate (AMP) (99%, Sigma), and phosphocreatine (PCr) (99%, Merck) were calculated according to the elution peak area and standard concentrations.

Sample ATP/ADP/AMP/PCr concentration (µg/L) = [sample peak area × standard ATP/ADP/AMP/PCr concentration]/peak area of the standard ATP/ADP/AMP/PCr concentration.

### Flow Cytometry Analysis of Cell Apoptosis

Five groups as mentioned earlier (see “Chemicals and Treatments” section) were investigated. Apoptotic rates were examined 48 h after the treatment. In accordance with the Annexin V/Propidium Iodide (PI) Apoptosis Kit (BioVision, CA, USA), 5 × 10^5^ cells were collected in each tube and 1 ml of annexin V binding buffer was added, followed by thorough mixing. Subsequently, 5 ml of annexin V–fluorescein isothiocyanate and 10 ml of PI were added. After mixing, the tube was incubated in the dark at 37°C for 15 min. For the early apoptotic cells, the membrane phosphatidylserine was exposed and combined with annexin V, without PI. For the late apoptotic cells, the membranes were permeable to PI and the cells were stained with annexin V and PI. The dead cells were stained only with PI. The samples were analyzed using a FACScan flow cytometer (BD Biosciences, NJ, USA) within 1 h. Flow cytometry (BD FACSAria; BD Biosciences) was performed to detect cell apoptosis.

### Western Blot Analysis

The primary antibodies to AMPK (1:1,000 dilution, sc-25792; Santa Cruz Biotechnology, Inc., TX, USA), p-AMPKα1/2 (Thr183/172) (1:1,000 dilution, sc-101630; Santa Cruz Biotechnology, Inc.), peroxisome proliferator-activated receptor-γ (PPAR-γ, 1:1,000 dilution, ab209350; Abcam, Cambridge, UK), CPT-1 (H40) (1:1,000 dilution, sc-98834; Santa Cruz Biotechnology, Inc.), GLUT-4 (1:500 dilution, bs-0384R; Bioss Antibodies, MA, USA), PGC-1α (1:500 dilution, bs-1832R; Bioss Antibodies), Bax (1:1,000 dilution, ab32503; Abcam), Bcl-2 (1:500 dilution, bs-0032R; Bioss Antibodies), caspase-3 (1:500 dilution, bs-2593R; Bioss Antibodies), and horseradish peroxidase (HRP) goat anti-rabbit (IgG) secondary antibody (1:5,000 dilution, BV-S8008; BIOVAL, CA, USA) were used. To document the loading controls, the membrane was reported with a primary antibody against anti-rabbit HRP secondary antibody. The signals were quantified by scanning densitometry and computer-assisted image analysis.

### Recombinant Lentivirus

The recombinant lentivirus plasmid expressing short hairpin (sh) RNA targeting both AMPKα1 and AMPKα2 (TGAATTAAATCCACAGAAA) and the control shRNA (ACGACGTCAGCTGGTGCATGT) were created as described earlier (Tangeman et al., 2012). Lentiviruses were packaged in rat neonatal cardiomyocytes.

### Statistical Analysis

The experimental results were presented as mean ± standard error. One-way analysis of variance was used to compare differences among the three groups, followed by the Bonferroni *post hoc* test for multiple comparisons. *P* values <0.05 were considered statistically significant. Statistical analyses were performed using GraphPad Prism 7.0 software (GraphPad Software Inc., CA, USA).

## Results

### Selection of SMI Concentration and Its Effects on Cell Viability

**Figure 1:**
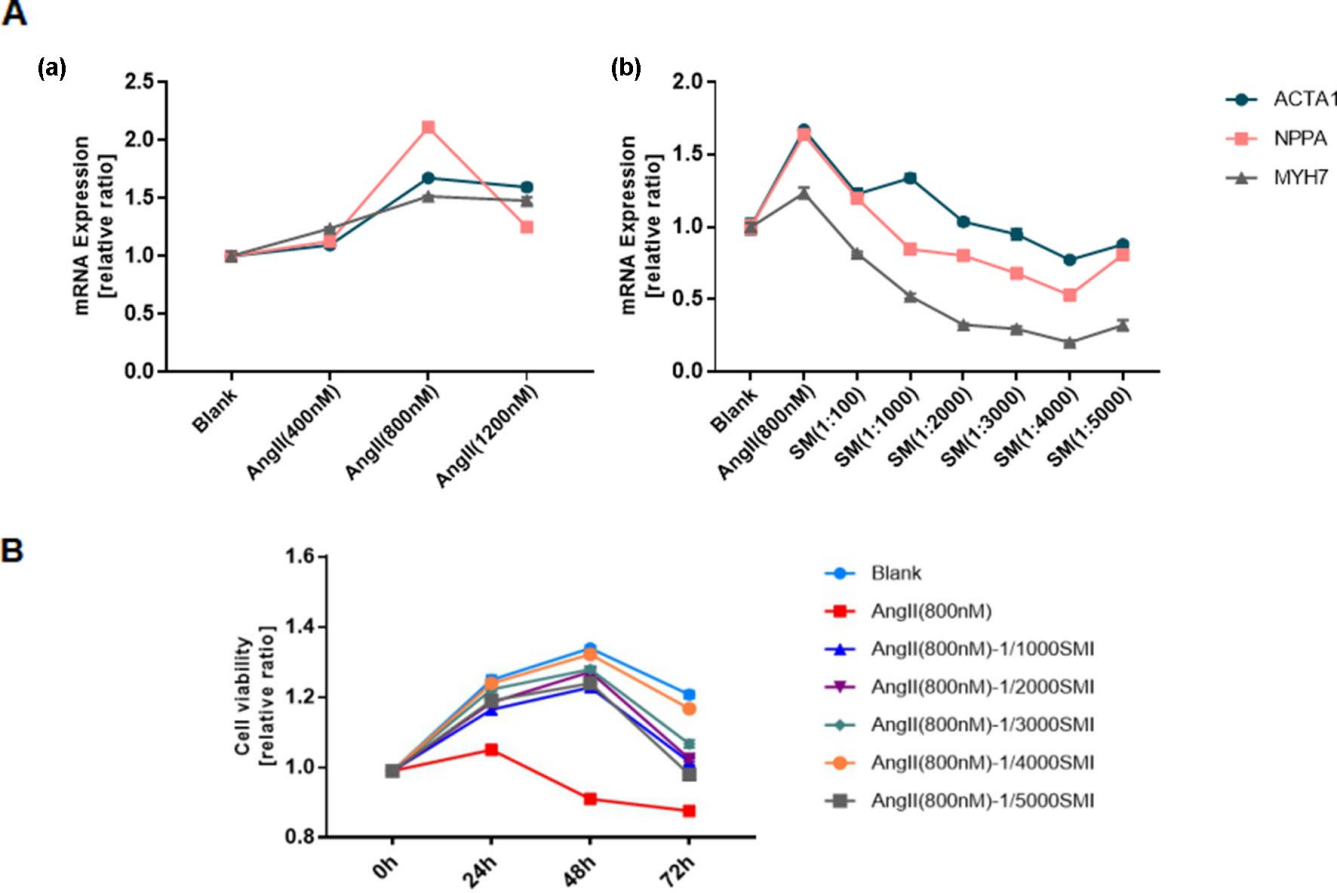
Selection of SMI concentration and its effects on cell viability. **(A)** (a) Selection of Ang II concentration (*n* = 3); (b) Selection of SMI concentration (*n* = 3). **(B)** Effects of SMI on cell viability at different time points and concentrations (*n* = 3).

Consistent with the findings of previous studies, cardiac hypertrophy could be induced by Ang II (Patrucco et al., 2014; Liu et al., 2015; Dong et al., 2017). RT-qPCR was used to quantify the RNA levels of the hypertrophic markers, such as ANP, BNP, and β-MHC, in cardiomyocytes to determine the concentrations of Ang II and SMI. For this purpose, the effects of Ang II (0.4, 0.8, and 1.2 µM) on the expression levels of these proteins in cardiomyocytes were first investigated. In addition, Ang II (0.8 µM) caused the maximum increase in the mRNA expression of ANP, BNP, and β-MHC compared with the blank group (**Figure 1A-a**). In contrast, 1/4,000 SMI significantly attenuated Ang II–induced expression levels of ANP, BNP, and β-MHC (**Figure 1A-b**). A sharp drop in NPPA after treatment with Ang II (1,200 nM) might be related to the cytotoxicity of high-dose Ang II, and NPPA was more sensitive.

To assess the effects of SMI on the viability of cardiomyocytes at different time points and concentrations, a CellTiter-Glo Luminescent Cell Viability Assay Kit (Promega, WI, USA) was used. The results showed that 1/4,000 SMI had the best cardiomyocyte viability after incubating with Ang II for 48 h (**Figure 1B**).

### SMI Attenuated Ang II–Induced Cardiac Hypertrophy in Rat Neonatal Cardiomyocytes

**Figure 2:**
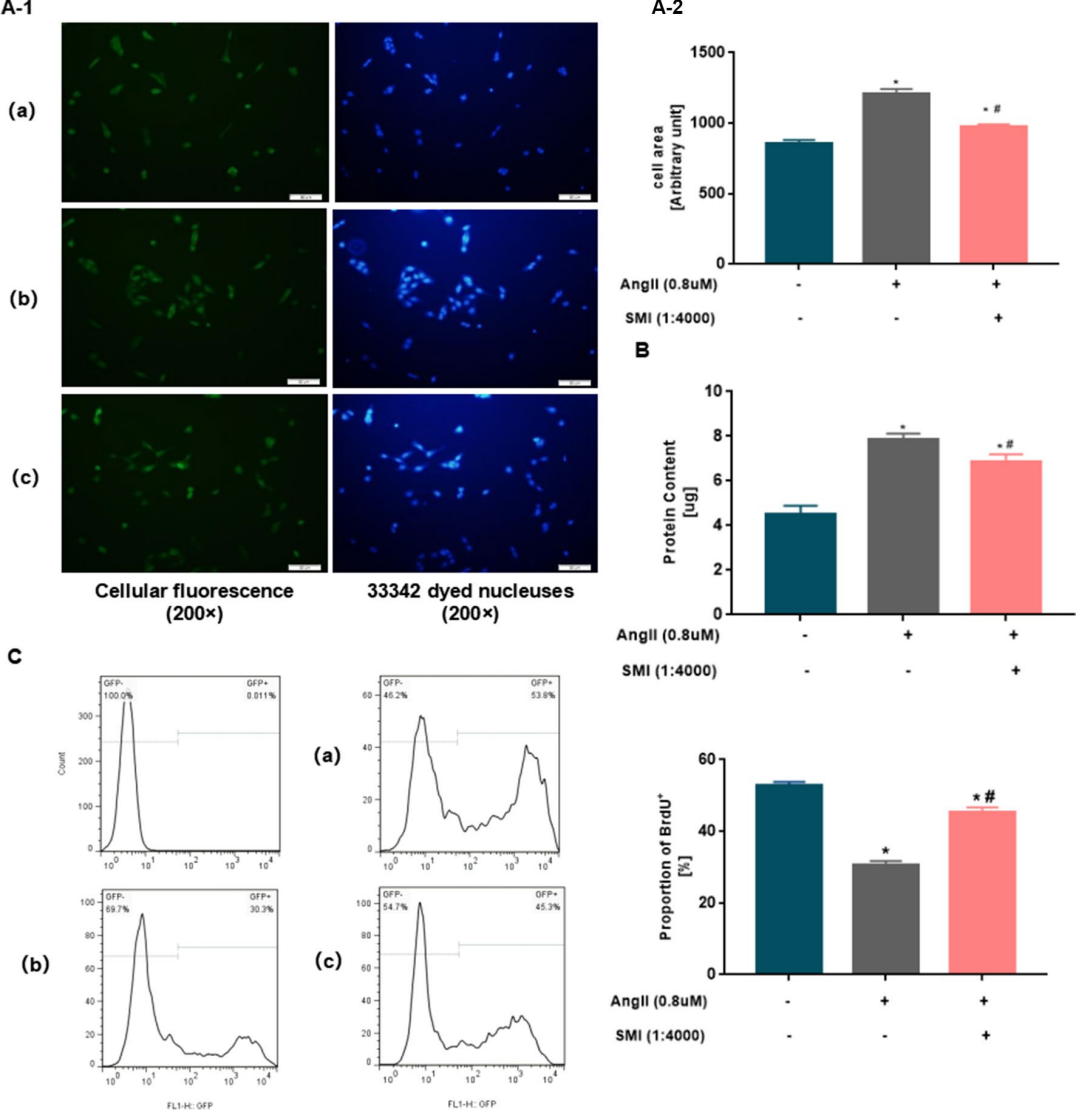
SMI attenuated Ang II–induced cardiac hypertrophy in rat neonatal cardiomyocytes. **(A)** Cell surface in three groups was assessed using an inverted microscope (200×). **(B)** Average protein content of cardiomyocytes in three groups was assessed using BCA. *P < 0.05, compared with the blank group; ^#^ *P* <0.05, compared with the Ang II group. **(C)** Percentages of BrdU^+^ cells assayed by BrdU incorporation. Numbers represent the percentage of BrdU^+^ cells in rat neonatal cardiomyocytes. *P < 0.05, compared with the blank group; ^#^ *P* <0.05, compared with the Ang II group. (a) Blank group, (b) Ang II group (Ang II 0.8µM), and (c) SMI group (Ang II 0.8µM + SMI 1/4,000).

To investigate whether SMI could attenuate Ang II–induced cardiac hypertrophy, neonatal mouse cardiomyocytes were treated with SMI (1/4,000) and/or Ang II (0.8 µM) as described in the Materials and Methods section. Ang II markedly increased the area of cardiomyocytes (*P* < 0.05), and SMI could reverse the effects of Ang II (*P* < 0.05) (**Figure 2A**).

To further investigate the effects of SMI on cardiac hypertrophy, the effects of SMI on the average protein content of cardiac hypertrophy were examined with BCA. Ang II significantly increased the average protein content of cardiomyocytes (*P* < 0.05), and SMI could reverse the effects of Ang II (*P* < 0.05) (**Figure 2B**).

Nascent DNA synthesis in replicating cells is traditionally measured with synthetic thymidine analogs such as BrdU. BrdU antibody staining showed a significant difference between BrdU nonproliferating cells and BrdU proliferating cells. Compared with the blank group, the proportion of BrdU^+^ in the Ang II group decreased significantly (*P* < 0.05). However, the SMI treatment group showed an improved increase (*P* < 0.05) (**Figure 2C**). Collectively, these observations indicated that the decreased average protein content of cardiomyocytes in the SMI group was not related to the inhibition of cardiomyocyte proliferation. Thus, SMI attenuated the hypertrophy of cardiomyocytes induced by Ang II.

### SMI Attenuated Mitochondria Dysfunction

**Figure 3:**
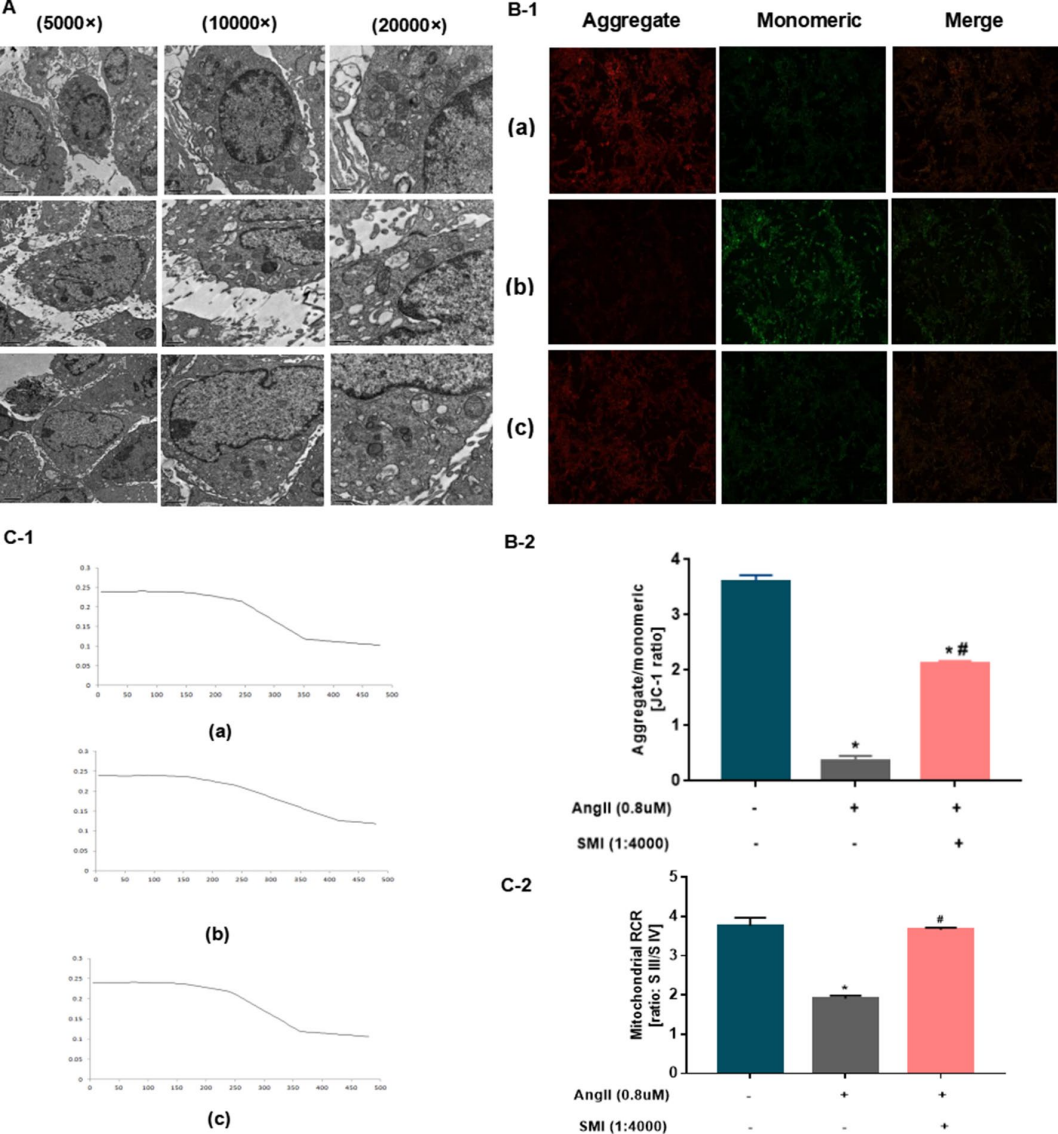
SMI had protective effects on mitochondria (*n* = 3). **(A)** Effects of SMI on the ultrastructural changes in cardiomyocytes and mitochondria assessed by TEM. **(B)** Effects of SMI on mitochondrial membrane potential assessed by JC-1 staining. **(C)** Effects of SMI on RCR assessed by dissolved oxygen electrolytic analyzer. *P < 0.05, compared with the blank group; ^#^ *P* < 0.05, compared with the Ang II group. (a) Blank group, (b) Ang II group (Ang II 0.8 uM), (c) SMI group (Ang II 0.8uM + SMI 1/4,000).

Next, the effects of SMI on the mitochondrial structure and function of myocardial cells were assessed. Ultrastructural changes were induced in cardiomyocytes and mitochondria in hypertrophic cardiomyocytes by Ang II treatment for 48 h, as detected by transmission electron microscopy. Compared with the blank group, the myocardial cell structure of the Ang II group was severely damaged, with several vacuole-like structures. The mitochondria became rounded and swollen, the sputum ruptured or even disappeared, the matrix became deep, some mitochondria dissolved and ruptured, and the membrane disappeared. The SMI group might improve the damage in mitochondrial structure to a certain degree (**Figure 3A**).

The mitochondrial membrane potential acts as an electrochemical gradient necessary to maintain mitochondrial structure and function and is capable of reflecting the mitochondrial functional status, which is also an indication of early apoptosis in cardiomyocytes. Thus, fluorescence was used to observe the effects of SMI on mitochondrial membrane potential. As shown in **Figure 3B**, when the heart function was normal, the mitochondrial membrane potential was relatively stable, with mostly red fluorescence. The myocardial cell mitochondria were damaged in the Ang II group, and the mitochondrial membrane potential notably decreased (*P* < 0.05). SMI played a significant role in stabilizing mitochondrial membrane potential (*P* < 0.05) (**Figure 3B**).

The RCR is one of the parameters reflecting mitochondrial function that can be used to measure mitochondria (Fatini et al., 2010). The myocardial mitochondrial RCR values were found to be significantly lower than those after HF, suggesting that the mitochondria’s ability to make use of oxygen was reduced. Compared with the blank group, the myocardial mitochondrial membrane potential declined in the Ang II group (*P* < 0.05). The SMI treatment group showed an improved mitochondrial respiratory function and increased RCR (*P* < 0.05) (**Figure 3C**).

### Changes in Expression Levels of ADP, ATP AMP, and PCr in Hypertrophic Cardiomyocytes

**Figure 4:**
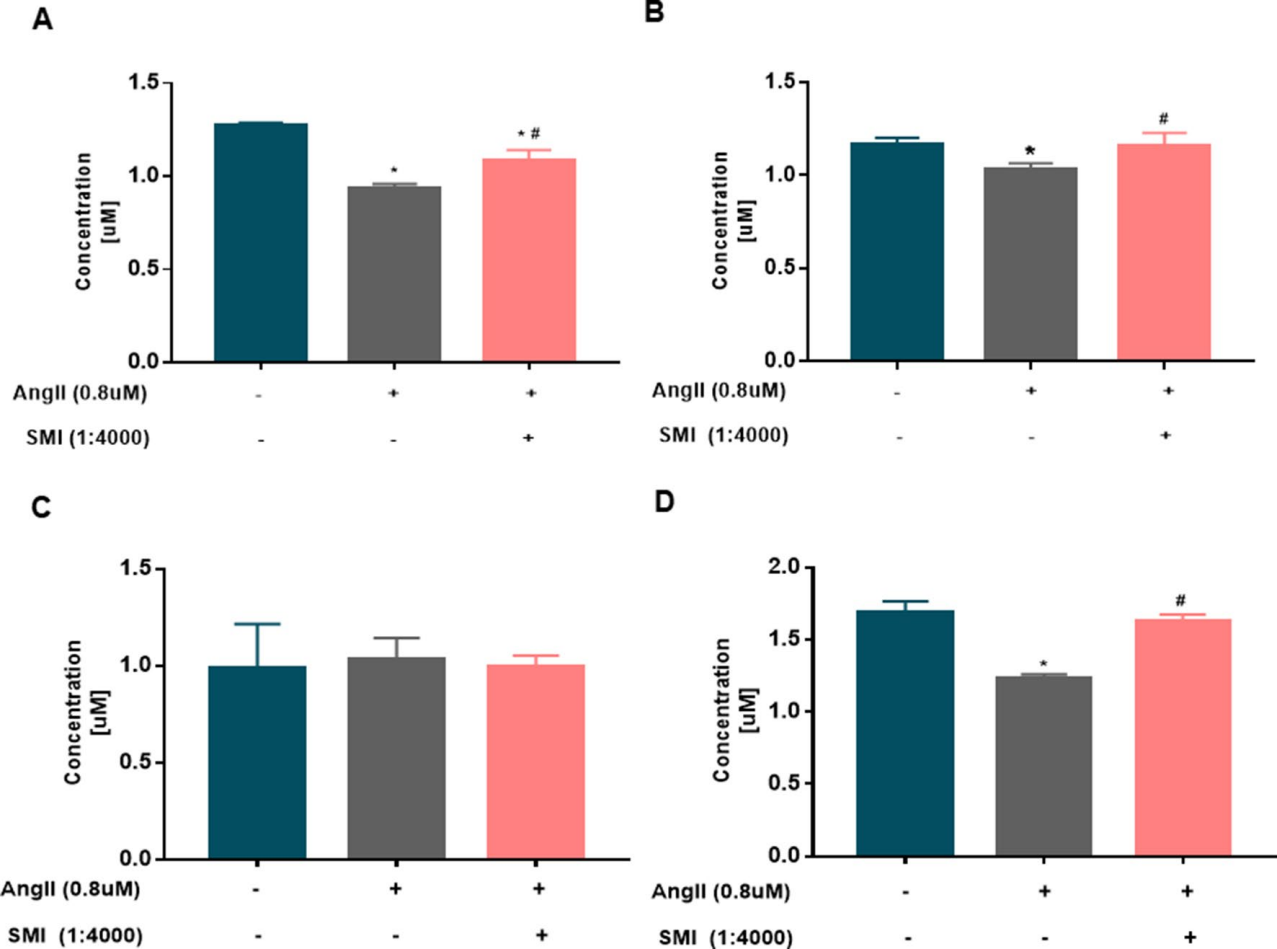
Changes in the levels of ADP, ATP, AMP, and PCr in hypertrophic cardiomyocytes (*n* = 6). **(A)** Changes in the ADP level in the three groups were assessed by HPLC. **(B)** Changes in the ATP level in the three groups were assessed by HPLC. **(C)** Changes in the AMP level in the three groups were assessed by HPLC. **(D)** Changes in the PCr level in the three groups were assessed by HPLC. *P < 0.05, compared with the blank group; ^#^ *P* < 0.05, compared with the Ang II group.

The continuous production of ATP by heart is a prerequisite for maintaining its own pump function, and ATP production is achieved mainly by oxidative phosphorylation in the mitochondrial electron transport chain. The energy in the cells is stored mainly in the form of PCr (Chen et al., 2012). Therefore, the energy level of cardiomyocytes and the function of mitochondria were measured by detecting the expression levels of ADP, ATP, AMP, and PCr *via* HPLC. Compared with the blank group, the expression levels of ATP, ADP, and PCr in the Ang II group were significantly lower (*P* < 0.05), revealing a disturbance in the supply of energy for cardiomyocytes in the Ang II group. Compared with the model group, the levels of ATP, ADP, and PCr remarkably increased in the SMI intervention group (*P* < 0.05). No significant difference was found in the expression level between the three groups (*P* > 0.05) (**Figures 4A–D**).

### SMI Regulates the Levels of Fatty Acid Metabolism, Glucose Oxidation, and Mitochondrial Biogenesis With CPT-1, GLUT-4, and PPARγ/PGC-1α Through the AMPK Signaling Pathway

**Figure 5:**
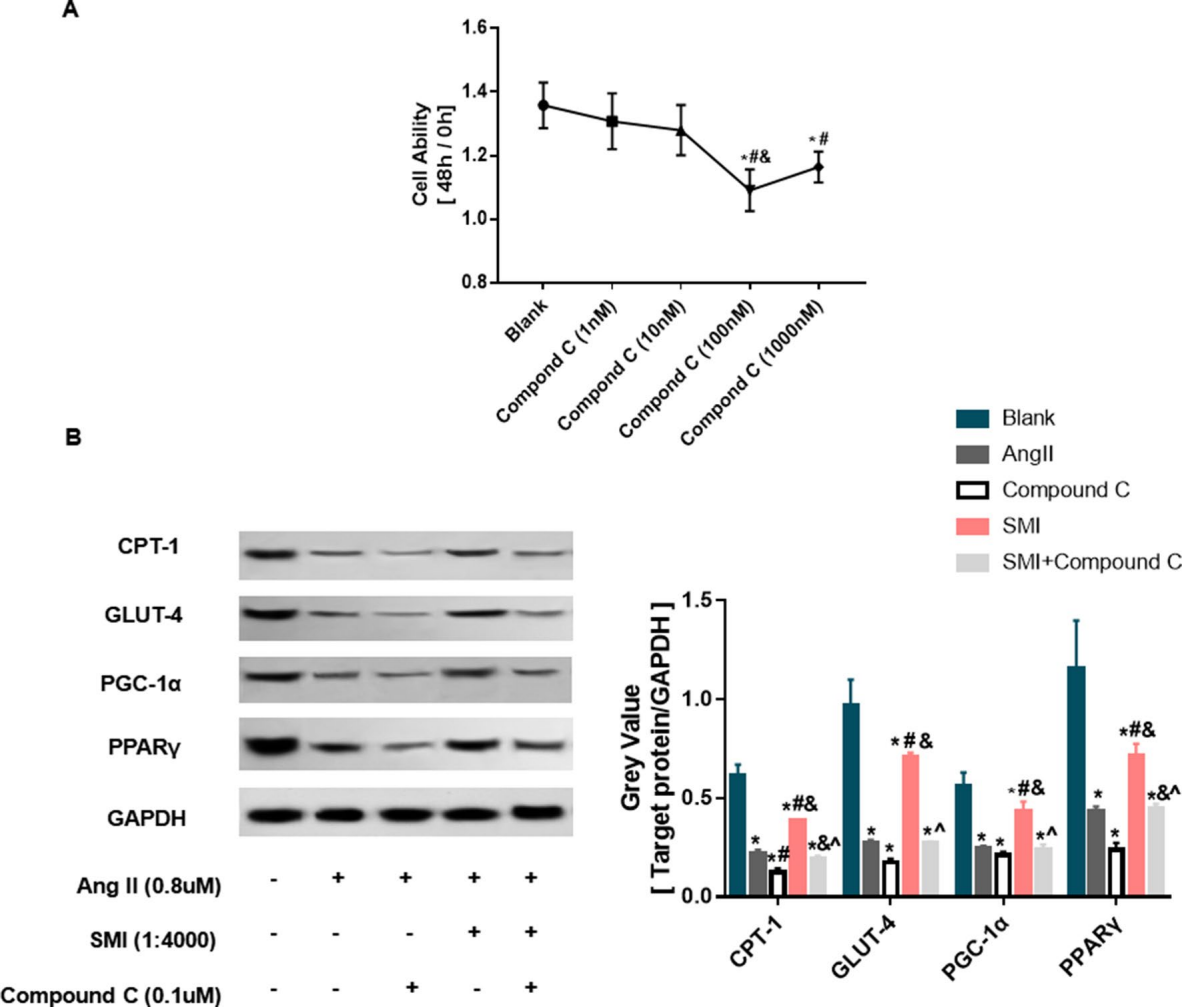
SMI regulates expression levels of CPT-1, GLUT-4, and PPAR-γ/PGC-1α through activating AMPK signaling pathway (*n* = 3). **(A)** Selection of AMPK concentration. *, compared with the blank group, ^#^ *P* < 0.05; compared with compound C (1 nM) group, ^&^ *p* < 0.05; compared with compound C (10 nM) group, *P* < 0.05. **(B)** SMI regulates protein expression levels of CPT-1, GLUT-4, and PPAR-γ/PGC-1α through activating AMPK signaling pathway. *, compared with the blank group, ^#^ *P* < 0.05; compared with Ang II group, ^&^ *p* < 0.05; compared with compound C group, ^*P* < 0.05; compared with SMI group, *P* < 0.05.

SMI was found to regulate the expression level of p-AMPK consistent with the expression trends of CPT-1, GLUT4, and PGC-1α (**Supplementary Figure 1**). Previous studies showed that AMPK could regulate the expression level of CPT-1 (Kim et al., 2017), GLUT-4 (Manna et al., 2017; Niu et al., 2017), and PGC-1α (Guo et al., 2018). Therefore, it was speculated that SMI regulated the levels of fatty acid metabolism, glucose oxidation, and mitochondrial synthesis probably by activating the AMPK signaling pathway. The effects of AMPK inhibitor compound C on cardiomyocyte viability were evaluated at different concentrations. When the concentration of compound C was 0.1µM, the cell viability was the worst after incubation with Ang II for 48 h (**Figure 5A**).

As illustrated in **Figure 5B**, after adding Ang II into cardiomyocytes, the expression level of CPT-1 protein significantly decreased compared with the control group (*P* < 0.05). During the process of Ang II–induced cardiac hypertrophy, the expression level of CPT-1 protein increased in the SMI group compared with the Ang II group. Compound C could reverse the effects of SMI (*P* < 0.05), indicating that SMI regulated CPT-1 *via* activating the AMPK signaling pathway. Consistent with fatty acid metabolism, the expression levels of GLUT-4 and PPARγ/PGC-1α proteins significantly decreased (*P* < 0.05) during Ang II–induced cardiomyocyte hypertrophy, but when SMI was added, these expression levels increased (*P* < 0.05). However, treating cells with AMPK inhibitor compound C notably decreased the expression levels of GLUT-4 and PPARγ/PGC-1α proteins (*P* < 0.05). These data indicated that SMI regulated the expression levels of CPT-1, GLUT-4, and PPARγ/PGC-1α *via* activating the AMPK signaling pathway.

### SMI Inhibited Apoptosis of Hypertrophic Cardiomyocytes *via* the AMPK Signaling Pathway

**Figure 6:**
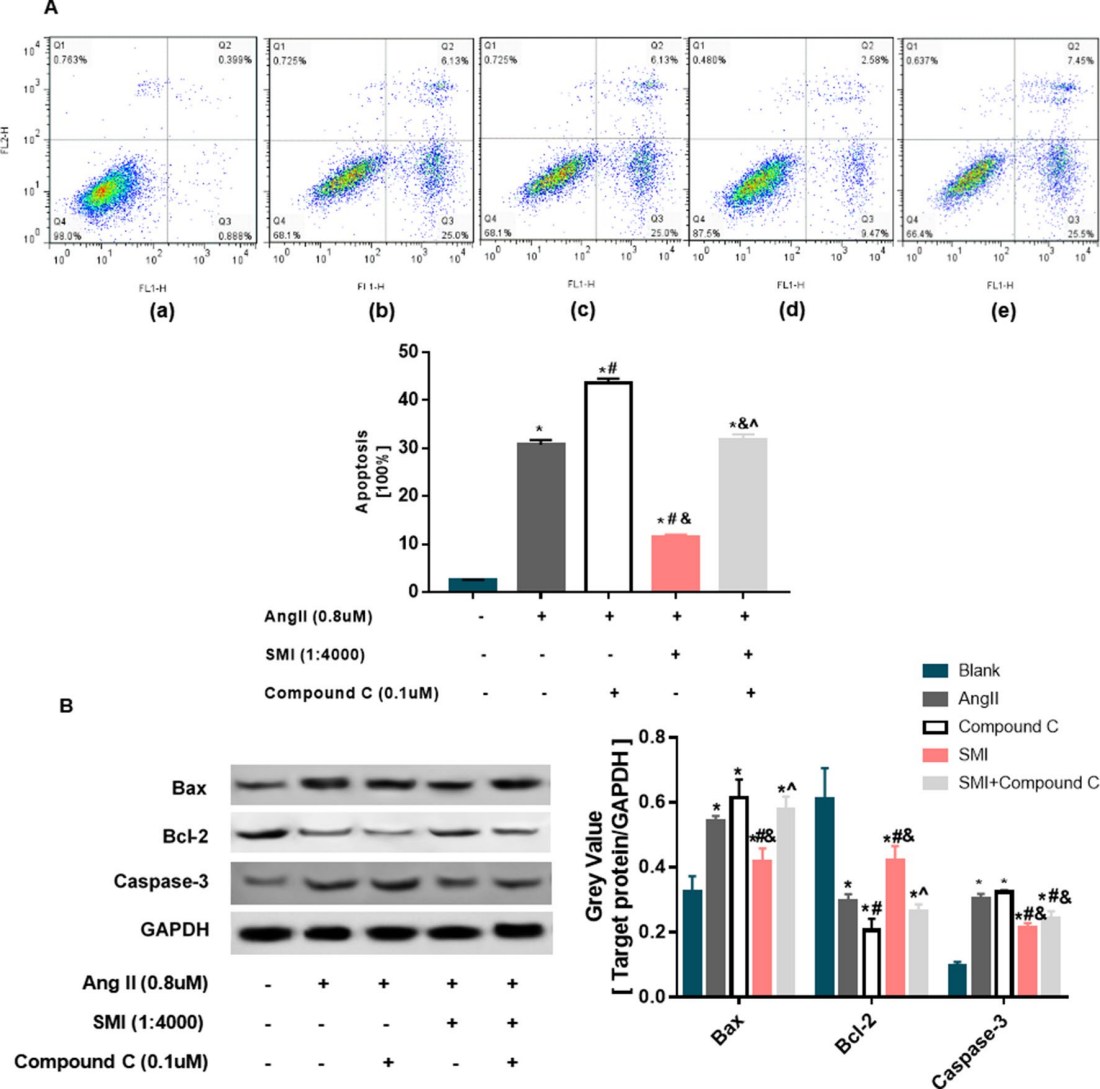
SMI inhibited the apoptosis of hypertrophic cardiomyocytes (*n* = 3). **(A)** Effects of SMI on cardiomyocyte apoptosis in hypertrophic cardiomyocytes. 3 Control group; (b) Ang II group; (c) compound C group; (d) SMI group; and (e) SMI + compound C group. Dead cells were labeled with annexin V (–) propidium iodide (PI) (+) and are shown in the Q1 area; late apoptotic cells were labeled with annexin V (+) PI (+) and are shown in the Q2 area; early apoptotic cells were labeled with annexin V (+) PI (–) and are shown in the Q3 area; live cells were labeled with annexin V (–) PI (–) and are shown in the Q4 area. Summarized data for TMRE fluorescence intensity measured with confocal microscopy. **(B)** Effects of SMI on apoptosis-related proteins in hypertrophic cardiomyocytes. *P < 0.05, compared with the blank group; ^#^ *P* < 0.05, compared with the Ang II group; ^&^ *P* < 0.05, compared with the compound C group; ^*P* < 0.05, compared with the SMI group.

Apoptosis is a key feature of the progression of heart diseases, and the elimination of pro-apoptotic stimulation or inhibition of the apoptotic cascade can rescue damaged myocardium and prevent the progression of adverse remodeling and HF (Abbate and Narula, 2012). Cardiomyocyte apoptosis may be the cause of cardiac hypertrophy to HF (Abbate et al., 2003). SMI could increase mitochondrial membrane potential (ΔΨm). However, the decline or disappearance of ΔΨm is an early event of myocardial cell apoptosis (Wlodkowic et al., 2012). Therefore, the effects of SMI on cardiomyocyte apoptosis were evaluated. Ang II markedly decreased the rate of apoptosis (*P* < 0.05), and compound C could reverse the protective effects of SMI on cardiomyocyte apoptosis partly (*P* < 0.05) (**Figure 6A**).

To further reveal the role of SMI in inhibiting cardiomyocyte apoptosis, the levels of apoptosis-related proteins were examined by Western blot analysis. As displayed in **Figure 6B**, compared with the blank group, the expression levels of Bax and caspase-3 proteins in the Ang II group were significantly higher, while the expression level of Bcl-2 was lower (*P* < 0.05), demonstrating that cardiomyocyte apoptosis occurred in the Ang II–induced cardiomyocyte hypertrophy group. Compared with the model group, the expression levels of Bax and caspase-3 increased and the expression level of Bcl-2 decreased in the SMI intervention group (*P* < 0.05). Compound C could reverse the effects of SMI on the expression levels of Bax and Bcl-2 (*P* < 0.05) (**Figure 6B**).

### Effect of AMPK on SMI-Mediated Cardiomyocyte Hypertrophy and Apoptosis

**Figure 7:**
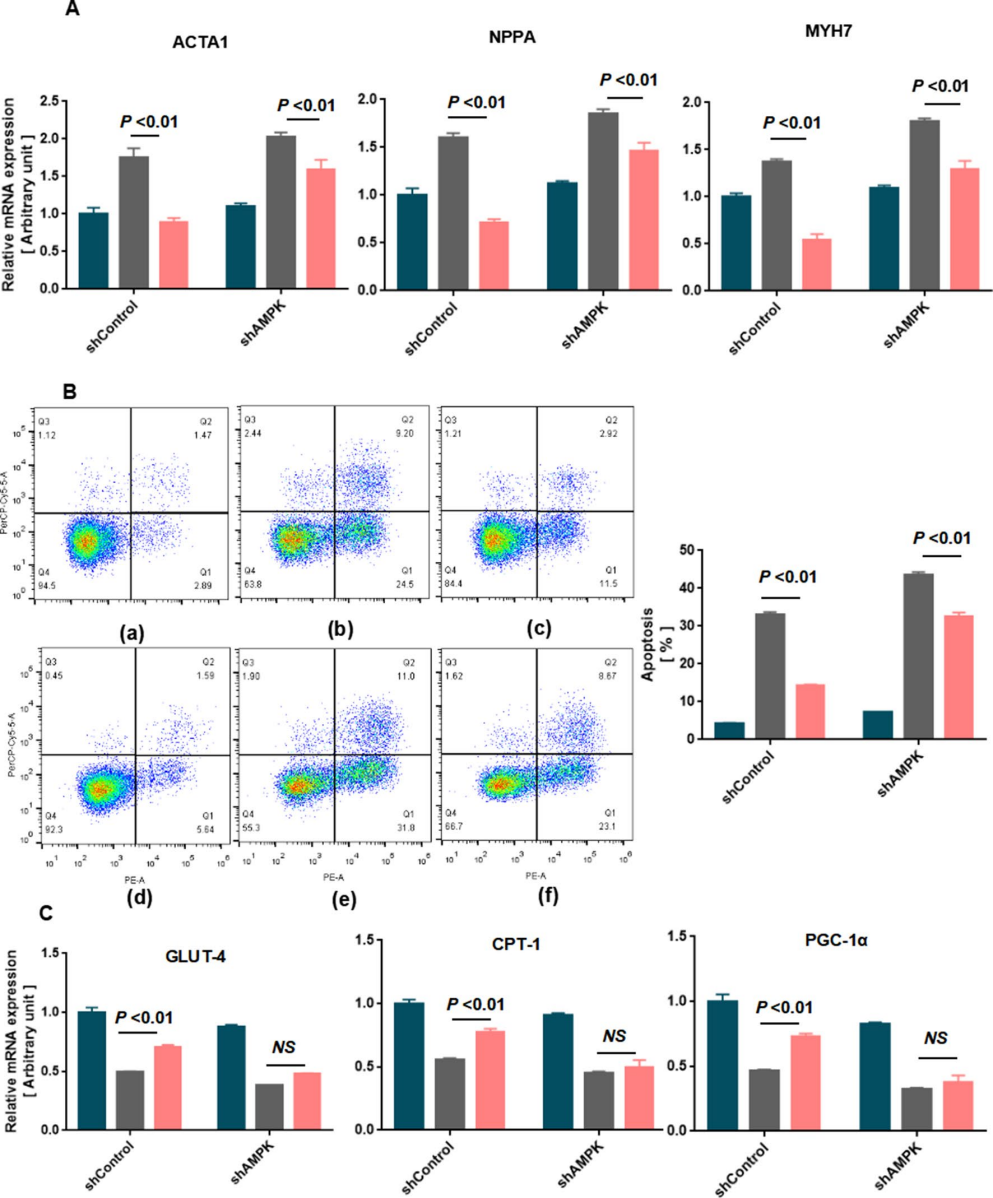
Knockdown of AMPK aggravated SMI-mediated cardiomyocyte hypertrophy and apoptosis in rat neonatal cardiomyocytes (*n* = 3). **(A)** Quantification of RT-qPCR was used to quantify the RNA levels of the hypertrophic markers in blank, Ang II-treated, or SMI-treated rat neonatal cardiomyocytes with shCtrol or shAMPK. **(B)** Annexin V/PI apoptosis kit was used to quantify cardiomyocyte apoptosis in blank, Ang II-treated, or SMI-treated rat neonatal cardiomyocytes with shCtrol or shAMPK. (a) Blank + shCtrol; (b) Ang II (0.8 µM) + shCtrol; (c) SMI (1/4,000) + Ang II (0.8 µM) + shCtrol; (d) Blank + shAMPK; (e) Ang II (0.8 µM) + shAMPK; (f) SMI (1/4,000) + Ang II (0.8 µM) + shCtrol. **(C)** Quantification of RT-qPCR was used to quantify the RNA levels of GLUT-4, CPT-1, and PGC-1α.

To further validate the effects of AMPK in inhibiting cardiomyocyte hypertrophy and apoptosis treated with SMI, AMPK knockdown experiments were performed using shRNA. Thus, lentivirus vectors expressing shRNA to both AMPK alpha1 and alpha2 were constructed (Tangeman et al., 2012) (**Supplementary Figure 4**). Decreased myocardial hypertrophy and apoptosis treated with SMI were partly inhibited by AMPK knockdown (**Figures 7A, B**). AMPK knockdown almost abolished the SMI-induced increase in the expression of GLUT-4, CPT-1, and PGC-1α (**Figure 7C**). The mechanism underlying increased levels of fatty acid metabolism, glucose oxidation, and mitochondrial biogenesis with CPT-1, GLUT-4, and PGC-1α was dependent on an SMI-mediated increase in AMPK expression. These observations corresponded with the experiments on AMPK inhibitor compound C.

## Discussion

The present study demonstrated that SMI not only reduced the cardiomyocyte hypertrophy and cell apoptosis induced by Ang II but also protected the mitochondrial function in hypertrophic cardiomyocytes. In addition, SMI improved energy metabolism by activating the expression levels of related factors in Ang II–induced cardiomyocyte hypertrophy, such as energy metabolism (AMPK), fatty acid metabolism (CPT-1), mitochondrial biogenesis factor (PGC-1α), and glucose oxidation (GLUT-4). These data suggested the phosphorylation of AMPK as a metabolic switch reconstituting fatty acid metabolism, glucose metabolism, and mitochondrial biogenesis to prevent cardiomyocyte hypertrophy and cell apoptosis. A previous study found that cardiac hypertrophy was both an intermediate step and a determinant of HF (Meijs et al., 2007). This was of great significance to identify alternative therapeutic approaches for HF.

The main source of normal adult myocardial energy is FFA oxidation. FFA oxidation disorder and increased glucose utilization occur in hypertrophic and failing myocardium, in which the direct consequence of this transformation is the reduction of fatty acid utilization. Although the oxidation of glucose needs less oxygen, it produces much less energy compared with FFA oxidation, causing the heart to remain in an energy-starved state, ultimately leading to a lack of myocardial energy. Due to the high energy demand of the heart, a fine balance in energy substrate (glucose and fatty acid) use is crucial in maintaining metabolic flexibility (Karwi et al., 2018). A key metabolic regulator is AMPK; a heterotrimeric protein is centrally involved in controlling cellular energy homeostasis. Its activation through metabolic stress stimulates energy-generating processes and has been shown to regulate hypertrophy-regulating genes in several models. The activation of the AMPK signaling pathway increases FFA oxidation by phosphorylating ACC, in turn decreasing the malonyl-CoA content and activating CPT-1. AMPK stimulates GLUT-4 translocation and increases the whole-body use of glucose (Yamaguchi et al., 2005). However, knowledge about the influence of Ang II on cardiac energy metabolism is limited. In the present study, when the action time reached 48 h, the expression levels of p-AMPK, CPT-1, and GLUT-4 decreased, suggesting that glucose metabolism and fatty acid metabolism were weakened. To validate further the effects of AMPK in SMI-mediated energy metabolism, AMPK knockdown experiments were performed using shRNA. The findings were consistent with the results of previous studies (Liu et al., 2018). Mitochondrial biogenesis is regulated by a complex regulatory system. PGC-1α, a transcription coactivator of nuclear receptors and master regulator of metabolism, plays a pivotal role in mediating metabolic alterations in both physiological and pathological hypertrophies. PGC-1α is also involved in mitochondrial biogenesis, which is vital for cell survival (Kulikova et al., 2018). The present study confirmed that SMI activated the AMPK signaling pathway and upregulated the expression levels of PGC-1α, CPT-1, and GLUT-4 compared with the model group. Thus, SMI regulated energy metabolism by activating the AMPK/PPARγ/PGC-1α signaling pathway. Specifically, SMI regulated fatty acid and glucose metabolism *via* activating the AMPK signaling pathway.

The energy metabolism in cardiomyocytes is closely associated with the status of mitochondrial function. Mitochondrial dysfunction can be caused by the intermediate metabolite accumulation in HF. In the present study, SMI was found to reduce mitochondrial structural damage, decrease respiratory function, and reduce membrane potential, which were induced by Ang II induction. The cardiac hypertrophic program was accompanied by progressive mitochondrial remodeling, and particularly by mitochondrial permeability transition pore opening (Javadov and Karmazyn, 2007; Javadov et al., 2009). When the membrane potential decreased the uncoupling of oxidative phosphorylation, ATP was depleted, and the number of oxygen radicals increased, leading to the irreversible apoptosis of cardiac myocytes. Cell proliferation, apoptosis, and cell hypertrophy are interrelated and interacted with each other during ventricular remodeling (Sun et al., 2013). Cardiomyocyte hypertrophy assembly leads to apoptosis, resulting in myocardial failure–induced cardiovascular disease. Thus, cardiac hypertrophy and apoptosis are closely related to cardiovascular diseases, such as HF (Freundlich et al., 2013). The expression levels of Bax and caspase-3 increased while the expression level of Bcl-2 decreased in the Ang II group, thus justifying the increase in the rate of apoptosis; this result was consistent with the finding of a previous study (Hutchinson et al., 2008; Day et al., 2011).

ATP is produced in mitochondria, and it needs to be continuously produced to maintain their function. Studies have shown that mitochondria in cardiomyocytes account for about one third of the total cardiomyocytes (Ventura-Clapier et al., 2011), indirectly indicating that the supply of myocardial energy is inseparable from mitochondria with normal function and structure. Mitochondrial dysfunction may affect the function of myocardium (Guzun et al., 2011). When cardiomyocytes were induced by Ang II, the mitochondrial structure, respiratory function, and membrane potential were notably damaged. Therefore, a decline in the ATP level was noted in the Ang II group. A study suggested that an increased intracellular AMP/ATP ratio was the primary mechanism for the activation of AMPK (Oakhill et al., 2011). Recent studies have shown other sensitive AMPK activators besides the activation of AMPK signaling pathway. Also, the deletion and decline in FDP can directly activate AMPK rather than being dependent on AMP (Kemp and Oakhill, 2017). The present study revealed for the first time that FBP was associated with the activation mechanism of AMPK, with no dependence on AMP and ADP. The AMP content did not change in Ang II–induced hypertrophic cardiomyocytes compared with the blank group, and SMI did not increase the expression level of AMP. Thus, it was suggested that SMI might directly activate the AMPK signaling pathway to increase the expression levels of ATP, ADP, and PCr in hypertrophic cardiomyocytes.

However, this study had many limitations. It did not deeply investigate the specific mechanism by which SMI regulated the mitochondrial function, glucose metabolism, and fatty acid metabolism. No animal experiments were conducted to verify the results. SMI itself is a compound preparation of TCM, which is extracted from three herbs: *Panax ginseng* C.A. Mey, *Ophiopogon japonicus* (Thunb.) Ker Gawl., and *Schisandra chinensis* (Turcz.) Baill. More than 28 ginsenosides have been extracted from ginseng, which could delay cardiac mitochondrial impairment (Scott et al., 2001; Wang and Ren, 2002). SMI was found to have more than 10 components using HPLC in the present study, including ginsenoside Rg1, ginsenoside Re, ginsenoside Rf, ginsenoside Rb1, ginsenoside Rc, ginsenoside Rh1, ginsenoside Rd, schisandrin, ginsenoside Rg5 (Rk1), and ginsenoside Rh3. Which specific component of SMI regulated energy metabolism was not studied. These issues will be addressed in future studies.

## Conclusions

**Figure 8:**
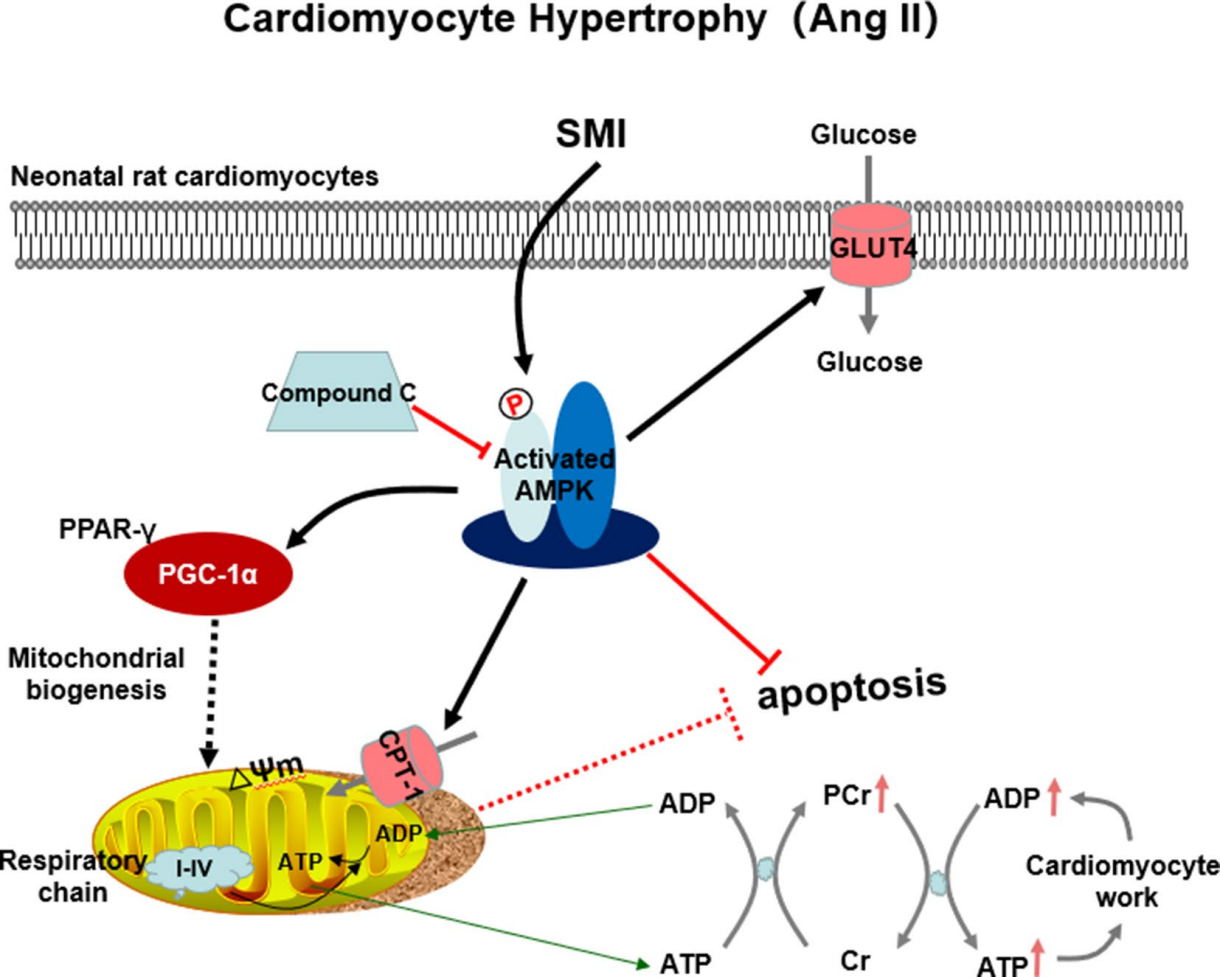
SMI suppressed Ang II–induced cardiomyocyte hypertrophy and apoptosis through the activation of the AMPK signaling pathway *via* energy-dependent mechanisms.

This study showed that SMI suppressed Ang II–induced cardiomyocyte hypertrophy and apoptosis through activating the AMPK signaling pathway *via* energy-dependent mechanisms (**Figure 8**). SMI can be considered to be an alternative therapeutic approach for HF.

## Data Availability

The datasets generated for this study can be found in the ANP: GenBank, NC_005104; BNP: GenBank, NC_005104;β-MHC: GenBank, NC_005114.

## Ethics Statement

This study was carried out in accordance with the principles of the Basel Declaration and recommendations of the Care and Use of Laboratory Animals Center of Shanghai University of Traditional Chinese Medicine. The protocol was approved by the ‘Experimental Animal Welfare and Ethics Committee of Shanghai University of Traditional Chinese Medicine’ (Permit No. PZSHUTCM190329013).

## Author Contributions

YL and XW designed the experiment, analyzed the data, and wrote the paper. YL, XR, XX, TQ and CL performed the experiments. HZ, XR and JG commented the manuscript. All authors have read and approved the manuscript.

## Funding

This study was supported by a grant from the National Natural Science Foundation of China (Grant No. 81803887, 81573647 and 81703743).

## Conflict of Interest Statement

The authors declare that the research was conducted in the absence of any commercial or financial relationships that could be construed as a potential conflict of interest.

## References

[B1] Abbate A. Biondi-Zoccai G. G. L. Bussani R. Dobrina A. Camilot D. Feroce F. (2003). Increased myocardial apoptosis in patients with unfavorable left ventricular remodeling and early symptomatic post-infarction heart failure. J. Am. Coll. Cardiol. 41 (5), 753–760. 10.1016/S0735-1097(02)02959-5 12628718

[B2] Abbate A. Narula J. (2012). Role of apoptosis in adverse ventricular remodeling. Heart Fail. Clin. 8 (1), 79–86. 10.1016/j.hfc.2011.08.010 22108728

[B3] Benjamin E. J. Muntner P. Alonso A. Bittencourt M. S. Callaway C. W. Carson A. P. (2019). Heart disease and stroke statistics—2019 update: a report from the american heart association. Circulation 139 (10), e56-e528. 10.1161/CIR.0000000000000659 30700139

[B4] Chen Y. Wang Y. Chen J. Chen X. Cao W. Chen S. (2012). Roles of transcriptional corepressor RIP140 and coactivator PGC-1α in energy state of chronically infarcted rat hearts and mitochondrial function of cardiomyocytes. Mol. Cell. Endocrinol. 362 (1-2), 11–18. 10.1016/j.mce.2012.03.023 22503866

[B5] Day R. M. Lee Y. H. Han L. Kim Y. C. Feng Y. H. (2011). Angiotensin II activates AMPK for execution of apoptosis through energy-dependent and -independent mechanisms. Am. J. Physiol. Lung Cell Mol. Physiol. 301 (5), L772–L781. 10.1152/ajplung.00072.2011 21856818 PMC3213991

[B6] De Jong K. A. Lopaschuk G. D. (2017). Complex energy metabolic changes in heart failure with preserved ejection fraction and heart failure with reduced ejection fraction. Can. J. Cardiol. 33 (7), 860–871. 10.1016/j.cjca.2017.03.009 28579160

[B7] Dolinsky V. W. Cole L. K. Sparagna G. C. Hatch G. M. (2016). Cardiac mitochondrial energy metabolism in heart failure: role of cardiolipin and sirtuins. Biochim. Biophys. Acta Mol. Cell Biol. Lipds. 1861 (10), 1544–1554. 10.1016/j.bbalip.2016.03.008 26972373

[B8] Dong Z. X. Wan L. Wang R. J. Shi Y. Q. Liu G. Z. Zheng S. J. (2017). (–)-Epicatechin suppresses angiotensin II-induced cardiac hypertrophy via the activation of the SP1/SIRT1 signaling pathway. Cell Physiol. Biochem. 41 (5), 2004–2015. 10.1159/000475396 28420000

[B9] Fan X. J. Yu H. Ren J. (2011). Homeostasis and compensatory homeostasis: bridging Western medicine and traditional Chinese medicine. Curr. Cardiol. Rev. 7 (1), 43–46. 10.2174/157340311795677671 22294974 PMC3131715

[B10] Fatini C. Sticchi E. Marcucci R. Verdiani V. Nozzoli C. Vassallo C. (2010). S38G single-nucleotide polymorphism at the KCNE1 locus is associated with heart failure. Heart Rhythm 7 (3), 363–367. 10.1016/j.hrthm.2009.11.032 20185111

[B11] Freundlich M. Li Y. C. Quiroz Y. Bravo Y. Seeherunvong W. Faul C. (2013). Paricalcitol downregulates myocardial renin-angiotensin and fibroblast growth factor expression and attenuates cardiac hypertrophy in uremic rats. Am. J. Hypertens. 27 (5), 720–726. 10.1093/ajh/hpt177 24072555 PMC3978945

[B12] Go A. S. Mozaffarian D. Roger V. L. Benjamin E. J. Berry J. D. Borden W. B. (2013). Executive summary: heart disease and stroke statistics—2013 update: a report from the American Heart Association. Circulation 127 (1), 143–152. 10.1161/CIR.0b013e318282ab8f 23283859

[B13] Guo X. Jiang Q. Tuccitto A. Chan D. Alqawlaq S. Won G.-J. (2018). The AMPK-PGC-1α signaling axis regulates the astrocyte glutathione system to protect against oxidative and metabolic injury. Neurobiol. Dis. 113, 59–69. 10.1016/j.nbd.2018.02.004 29438738

[B14] Guzun R. Timohhina N. Tepp K. Gonzalez-Granillo M. Shevchuk I. Chekulayev V. (2011). Systems bioenergetics of creatine kinase networks: physiological roles of creatine and phosphocreatine in regulation of cardiac cell function. Amino Acids 40 (5), 1333–1348. 10.1007/s00726-011-0854-x 21390528

[B15] Haynes P. Campbell K. S. (2014). Myocardial hypertrophy reduces transmural variation in mitochondrial function. Front. Physiol. 5, 178. 10.3389/fphys.2014.00178 24847280 PMC4019838

[B16] Hoppel C. L. Tandler B. Fujioka H. Riva A. (2009). Dynamic organization of mitochondria in human heart and in myocardial disease. Int. J. Biochem. Cell Biol. 41 (10), 1949–1956. 10.1016/j.biocel.2009.05.004 19446651 PMC3221317

[B17] Hutchinson D. S. Summers R. J. Bengtsson T. (2008). Regulation of AMP-activated protein kinase activity by G-protein coupled receptors: potential utility in treatment of diabetes and heart disease. Pharmacol. Ther. 119 (3), 291–310. 10.1016/j.pharmthera.2008.05.008 18606183

[B18] Javadov S. Karmazyn M. (2007). Mitochondrial Permeability transition pore opening as an endpoint to initiate cell death and as a putative target for cardioprotection. Cell Physiol. Biochem. 20, 01–22. 10.1159/000103747 17595511

[B19] Javadov S. Rajapurohitam V. Kilić A. Zeidan A. Choi A. Karmazyn M. (2009). Anti-hypertrophic effect of NHE-1 inhibition involves GSK-3β-dependent attenuation of mitochondrial dysfunction. J. Mol. Cell Cardiol. 46 (6), 998–1007. 10.1016/j.yjmcc.2008.12.023 19318234

[B20] Karwi Q. G. Uddin G. M. Ho K. L. Lopaschuk G. D. (2018). Loss of metabolic flexibility in the failing heart. Front. Cardiovasc. Med. 5, 68. 10.3389/fcvm.2018.00068 29928647 PMC5997788

[B21] Kemp B. E. Oakhill J. S. (2017). Energy sensing through a sugar diphosphate. Nature 548 (7665), 36–37. 10.1038/nature23099 28723890

[B22] Kim B. Woo M.-J. Park C.-S. Lee S.-H. Kim J.-S. Kim B. (2017). Hovenia Dulcis extract reduces lipid accumulation in oleic acid-induced steatosis of Hep G2 cells *via* activation of AMPK and PPARα/CPT-1 pathway and in acute hyperlipidemia mouse model. Phytother. Res. 31 (1), 132–139. 10.1002/ptr.5741 27762456

[B23] Kulikova T. G. Stepanova O. V. Voronova A. D. Valikhov M. P. Sirotkin V. N. Zhirov I. V. (2018). Pathological remodeling of the myocardium in chronic heart failure: role of PGC-1α. Bull. Exp. Biol. Med. 164 (6), 794–797. 10.1007/s10517-018-4082-1 29658071

[B24] Liu B. L. Cheng M. Hu S. Wang S. Wang L. Hu Z. Q. (2018). Effect of the Shensong Yangxin Capsule on energy metabolism in angiotensin ii-induced cardiac hypertrophy. Chin. Med. J. (Engl.) 131 (19), 2287–2296. 10.4103/0366-6999.241819 30246714 PMC6166447

[B25] Liu L. Wang C. Sun D. Jiang S. Li H. Zhang W. (2015). Calhex 231 Ameliorates cardiac hypertrophy by inhibiting cellular autophagy in Vivo and in Vitro. Cell Physiol. Biochem. 36 (4), 1597–1612. 10.1159/000430322 26159880

[B26] Luo Y. Xu Y. Liang C. Xing W. Zhang T. (2018). The mechanism of myocardial hypertrophy regulated by the interaction between mhrt and myocardin. Cell Signalling 43, 11–20. 10.1016/j.cellsig.2017.11.007 29199045

[B27] Manna P. Achari A. E. Jain S. K. (2017). Vitamin D supplementation inhibits oxidative stress and upregulate SIRT1/AMPK/GLUT4 cascade in high glucose-treated 3T3L1 adipocytes and in adipose tissue of high fat diet-fed diabetic mice. Arch. Biochem. Biophys. 615, 22–34. 10.1016/j.abb.2017.01.002 28063949

[B28] Meijs M. F. de Windt L. J. de Jonge N. Cramer M. J. Bots M. L. Mali W. P. (2007). Left ventricular hypertrophy: a shift in paradigm. Curr. Med. Chem. 14, 157–171. 10.2174/092986707779313354 17266575

[B29] Niu Y. Wang T. Liu S. Yuan H. Li H. Fu L. (2017). Exercise-induced GLUT4 transcription *via* inactivation of HDAC4/5 in mouse skeletal muscle in an AMPKα2-dependent manner. Biochim. Biophys. Acta Mol. Basis Dis. 1863 (9), 2372–2381. 10.1016/j.bbadis.2017.07.001 28688716

[B30] Oakhill J.S. Steel R. Chen Z. P. Scott J. W. Ling N. Tam S. (2011). AMPK is a direct adenylate charge-regulated protein kinase. Science 322 (6036), 1433–1435. 10.1126/science.1200094 21680840

[B31] Opie L. H. Commerford P. J. Gersh B. J. Pfeffer M. A. (2006). Controversies in ventricular remodelling. Lancet 367 (9507), 356–367. 10.1016/S0140-6736(06)68074-4 16443044

[B32] Patrucco E. Domes K. Sbroggio M. Blaich A. Schlossmann J. Desch M. (2014). Roles of cGMP-dependent protein kinase I (cGKI) and PDE5 in the regulation of Ang II-induced cardiac hypertrophy and fibrosis. Proc. Natl. Acad. Sci. 111 (35), 12925–12929. 10.1073/pnas.1414364111 25139994 PMC4156763

[B33] Quaini F. Chen Y. Tang Y. Zhang Y.-C. Huang X.-H. Xie Y.-Q. (2015). A Metabolomic study of rats with doxorubicin-induced cardiomyopathy and Shengmai injection treatment. PloS One 10 (5), e0125209. 10.1371/journal.pone.0125209 25938766 PMC4418690

[B34] Roman M. J. Ganau A. Saba P. S. Devereux R. B. (1997). Left ventricular hypertrophy, arterial compliance, and aging. Adv. Exp. Med. Biol. 432, 13–22. 10.1007/978-1-4615-5385-4_2 9433507

[B35] Rosano G. M. Vitale C. (2018). Metabolic modulation of cardiac metabolism in heart failure. Cardiac Fail. Rev. 4 (2), 99–103. 10.15420/cfr.2018.18.2 PMC6125709 30206484

[B36] Scott G. I. Colligan P. B. Ren B. H. Ren J. (2001). Ginsenosides Rb1 and Re decrease cardiac contraction in adult rat ventricular myocytes: role of nitric oxide. Br. J. Pharmacol. 134 (6), 1159–1165. 10.1038/sj.bjp.0704377 11704635 PMC1573065

[B37] Shah A. M. Shin S. H. Takeuchi M. Skali H. Desai A. S. Kober L. (2012). Left ventricular systolic and diastolic function, remodelling, and clinical outcomes among patients with diabetes following myocardial infarction and the influence of direct renin inhibition with aliskiren. Eur. J. Heart Fail. 14 (2), 185–192. 10.1093/eurjhf/hfr125 21965526

[B38] Sun B. Huo R. Sheng Y. Li Y. Xie X. Chen C. (2013). Bone morphogenetic protein-4 mediates cardiac hypertrophy, apoptosis, and fibrosis in experimentally pathological cardiac hypertrophy. Hypertension 61 (2), 352–360. 10.1161/HYPERTENSIONAHA.111.00562 23248151

[B39] Tamura T. Said S. Harris J. Lu W. Gerdes A. M. (2000). Reverse remodeling of cardiac myocyte hypertrophy in hypertension and failure by targeting of the renin-angiotensin system. Circulation 102 (2), 253–259. 10.1161/01.CIR.102.2.253 10889139

[B40] Tham Y. K. Bernardo B. C. Ooi J. Y. Y. Weeks K. L. McMullen J. R. (2015). Pathophysiology of cardiac hypertrophy and heart failure: signaling pathways and novel therapeutic targets. Arch. Toxicol. 89 (9), 1401–1438. 10.1007/s00204-015-1477-x 25708889

[B41] Tangeman L. Wyatt C. N. Brown T. L. (2012). Knockdown of AMP-activated protein kinase alpha 1 and alpha 2 catalytic subunits. J. RNAi Gene Silencing 8 (1), 470–478. 23316259 PMC3542724

[B42] Torrens C. Ghnenis A. B. Odhiambo J. F. McCormick R. J. Nathanielsz P. W. Ford S. P. (2017). Maternal obesity in the ewe increases cardiac ventricular expression of glucocorticoid receptors, proinflammatory cytokines and fibrosis in adult male offspring. PloS One 12 (12), e0189977. 10.1371/journal.pone.0189977 29267325 PMC5739430

[B43] Ventura-Clapier R. Garnier A. Veksler V. Joubert F. (2011). Bioenergetics of the failing heart. Biochim. Biophys. Acta Mol. Cell Res. 1813 (7), 1360–1372. 10.1016/j.bbamcr.2010.09.006 20869993

[B44] Wang Z. G. Ren J. (2002). Current status and future direction of Chinese herbal medicine. Trends Pharmacol. Sci. 23 (8), 347–348. 10.1016/S0165-6147(02)02051-5 12377568

[B45] Wlodkowic D.I. Skommer J. Darzynkiewicz Z. (2012). Cytometry of apoptosis. Historical perspective and new advances. Exp. Oncol. 34 (3), 255–262. 23070010 PMC3476471

[B46] Xian S. Yang Z. Lee J. Jiang Z. Ye X. Luo L. (2016). A randomized, double-blind, multicenter, placebo-controlled clinical study on the efficacy and safety of Shengmai injection in patients with chronic heart failure. J. Ethnopharmacol. 186, 136–142. 10.1016/j.jep.2016.03.066 27045864

[B47] Yamaguchi S. Katahira H. Ozawa S. Nakamichi Y. Tanaka T. Shimoyama T. (2005). Activators of AMP-activated protein kinase enhance GLUT4 translocation and its glucose transport activity in 3T3-L1 adipocytes. Am. J. Physiol.-Endocrinol. Metab. 289 (4), E643–E649. 10.1152/ajpendo.00456.2004 15928020

[B48] Zhan S. Fan X. Zhang F. Wang Y. Kang L. Li Z. (2015). A proteomic study of Shengmai injection’s mechanism on preventing cardiac ischemia-reperfusion injury *via* energy metabolism modulation. Mol. BioSyst. 11 (2), 540–548. 10.1039/C4MB00161C 25427756

[B49] Zhou Q. Qin W.-Z. Liu S.-B. Kwong J. S. W. Zhou J. Chen J. (2014). Shengmai (a traditional Chinese herbal medicine) for heart failure. Cochrane Database Syst. Rev. (4), CD005052. 10.1002/14651858.CD005052.pub5 24733159

