# Supplementary material for: Shengmai Injection Suppresses Angiotensin II-Induced Cardiomyocyte Hypertrophy and Apoptosis via Activation of the AMPK Signaling Pathway Through Energy-Dependent Mechanisms
Source: Front Pharmacol. 2019 Sep 20;10:1095. doi: 10.3389/fphar.2019.01095 (PMC6764192; doi:10.3389/fphar.2019.01095)
Supplement: Supplementary file 1 [file DataSheet_1.pdf]

Supplementary Material

1 Supplementary Data

Pre-experiment of **FIGURE 5** in the manuscript.

SMI regulates energy balance, fatty acids, glucose oxidation, and mitochondrial biogenesis with p-AMPK, CPT-1, GLUT-4, PGC-1α

To explore the effects of SMI on energy balance, fatty acids, glucose oxidation, and mitochondrial biogenesis, we examined the protein expression of the energy metabolism factor – AMPK, fatty acid and glucose oxidation key factors – CPT-1 and GLUT-4 and mitochondrial biogenesis factor – PGC-1α by western bolt (WB) assay. As shown in Figure 3, Compared with the blank group, the protein expression of p-AMPK, CPT-1, GLUT-4 and PGC-1α was declined in the Ang II group. SMI treatment group could reverse the effects of Ang II.

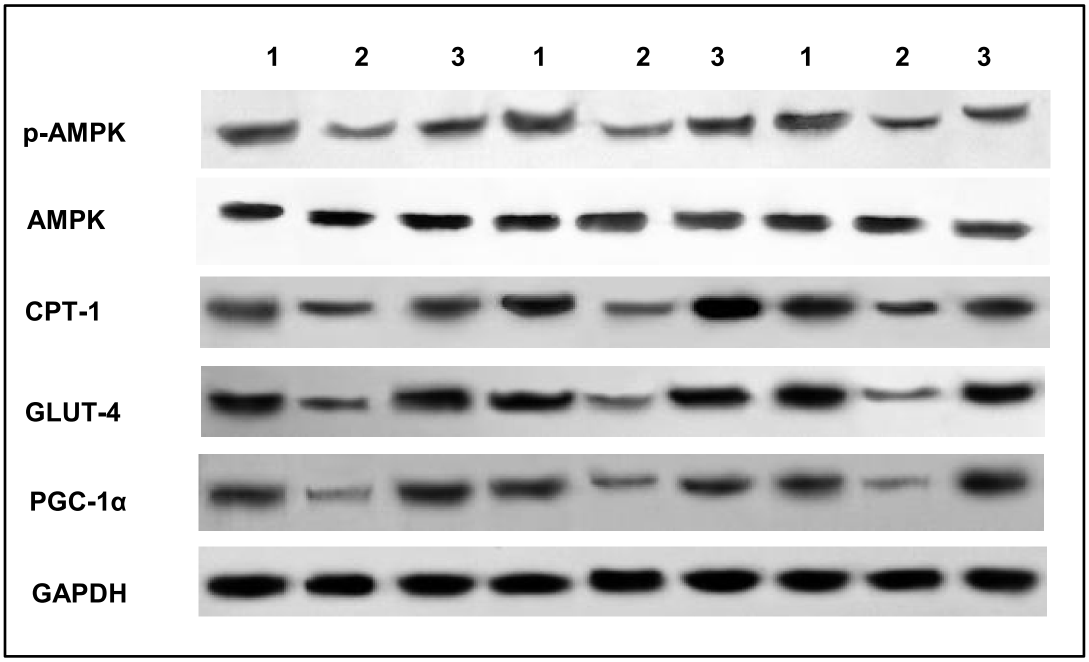

Supplementary Figure 1. SMI regulates energy balance, fatty acids, glucose oxidation, and mitochondrial biogenesis with p-AMPK, CPT-1, GLUT-4, PGC-1α. 1. Blank group; 2. Ang II group; 3. SMI group. p-AMPK: Phosphorylation AMP-activated protein kinase; AMPK: AMP-activated protein kinase; CPT-1: Carnitine acyltransferase enzyme-1; GLUT-4: Glucose transporter protein-4; PGC-1α: Peroxisome proliferator-activated receptor γ coactivator-1α.

2 Supplementary Figures and Tables

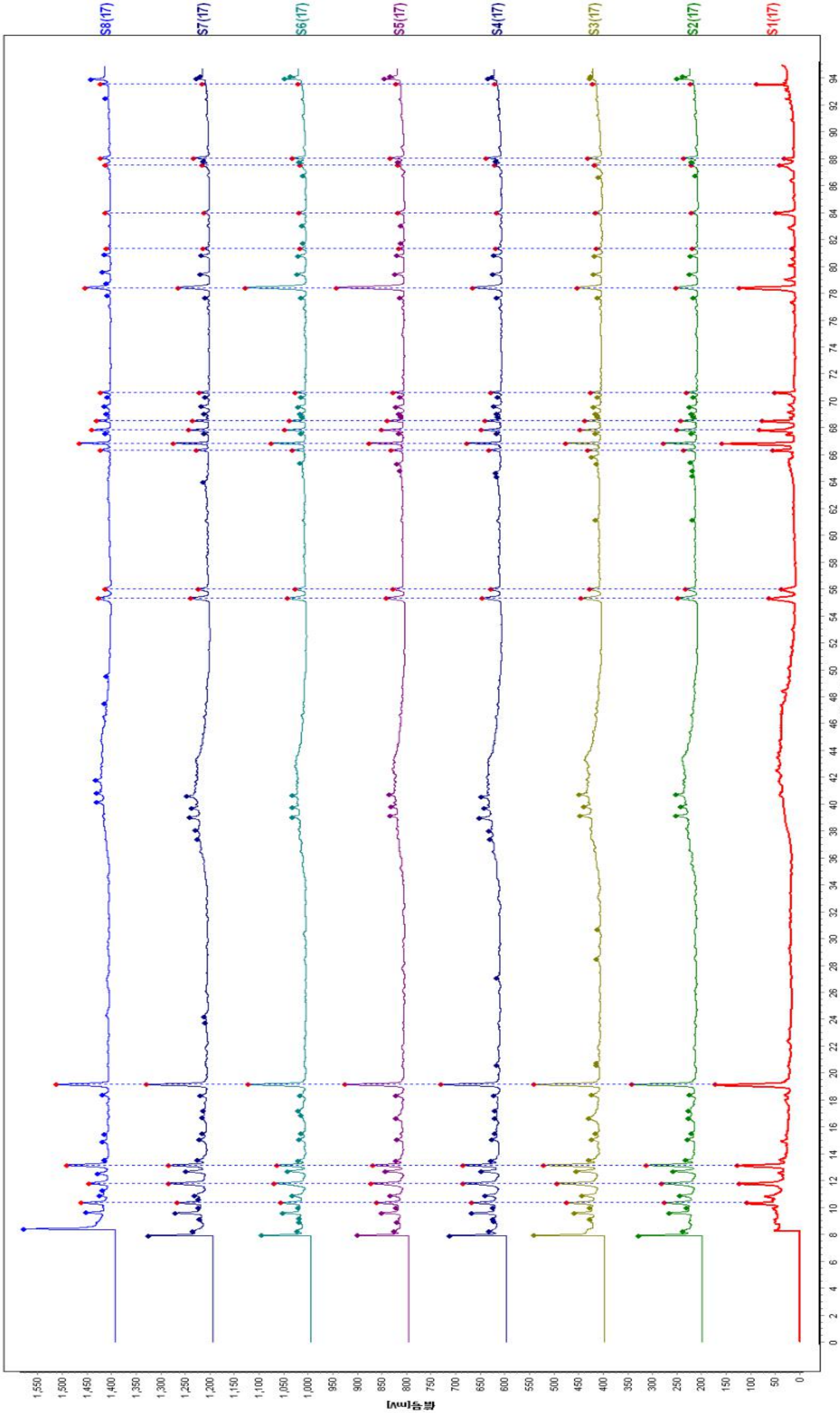
S8(17)
S7(17)
S6(17)
S5(17)
S4(17)
S3(17)
S2(17)
S1(17)
时间[min]
信号[mV]

Supplementary Figure 2. Fingerprint of Shengmai Injection. S1: National standard (China); S2-S7: H18050201, H18050202, H18050301, H18050302, H18050401, H18050402; S8: H17050402 (Production batch number of SMI in this experiment).

Production method of SMI

After the extraction, concentration and purification of 100g red ginseng (*Panax ginseng* C.A.Mey. after processing), add aquae pro injectione (170512, 170515, 170517, 170519 and 170522; Shanghai Hutchison Pharmaceuticals, Shanghai, China) to 200ml, then made it into red ginseng precipitate solution through a series of processes.

312g *Ophiopogon japonicus* (Thunb.) Ker Gawl.was extracted, concentrated and purified by adding aquae pro injectione to 200ml, then made it into ophiopogon japonicus precipitate solution.

Collect 150ml *Schisandra chinensis* (Turcz.) Baill. distilled liquid by 156g Schisandra chinensis . The residue was extracted, concentrated and purified. After adding aquae pro injectione to 150ml, Schisandra chinensis precipitate solution was prepared by a series of processes.

The above red ginseng precipitate solution (200ml), ophiopogon japonicus precipitate solution (200ml), schisandrae chinensis distillate (150ml) and schisandrae chinensis precipitate solution (200ml) were combined. After the purification process, then add aquae pro injectione to 1000 ml.

Supplementary Table 1. Raw material information of SMI (H17050402)

Raw material	Lot number	Supplier	Content
red ginseng (*Panax ginseng* C.A.Mey. after processing)	H20140901<br>H20150903<br>H20150903-1	Jiju Shenye Co., Ltd., China	100g
Ophiopogon japonicus (Thunb.) Ker Gawl.	H2016090802	SPH Huayu Chinese Herbs, China	312g
Schisandra chinensis (Turcz.) Baill.	H2016101705-1<br>H2016111602	SPH Huayu Chinese Herbs, China	156g
			Made into 1000ml

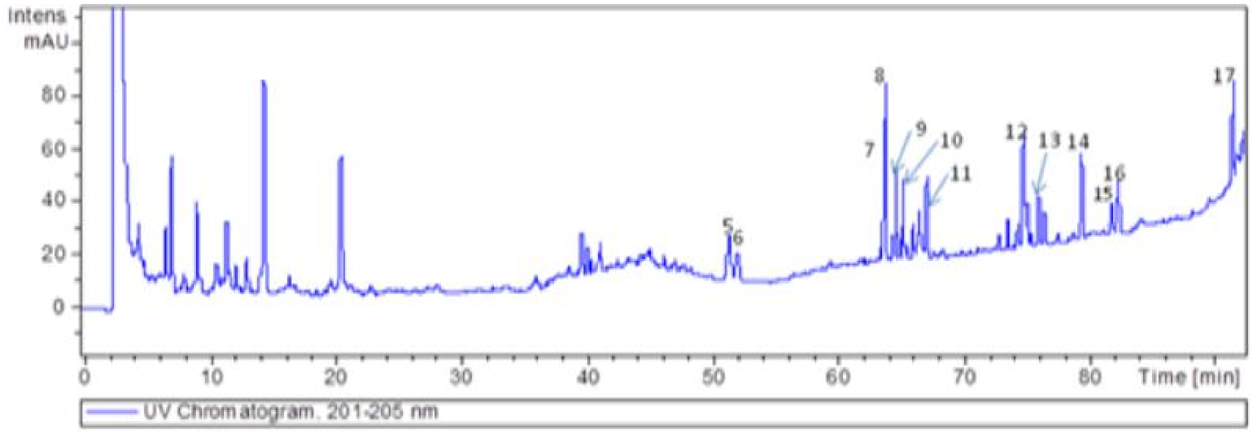

Supplementary Figure 3. Characteristic peaks of SMI assay by HPLC. 5: ginsenoside Rg1, 6: ginsenoside Re, 7: ginsenoside Rf, 8: ginsenoside Rb1, 9: ginsenoside Rc, 10: ginsenoside Rh1, 11: ginsenoside Rd, 12: schisandrin, 14: unknown (278 kD), 15 (16): ginsenoside Rg5 (ginsenoside Rk1), 17: ginsenoside Rh3.

Supplementary Table 2. Concentrations of major components in SMI

Number of peak	Structure	Component	R1	R2	Concentration (ug/ml)
5	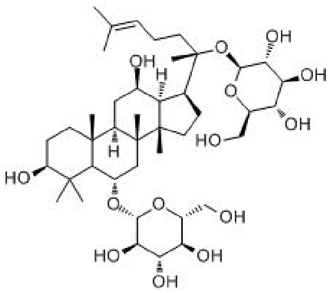	ginsenoside Rg1	Glc	Glc	295
6	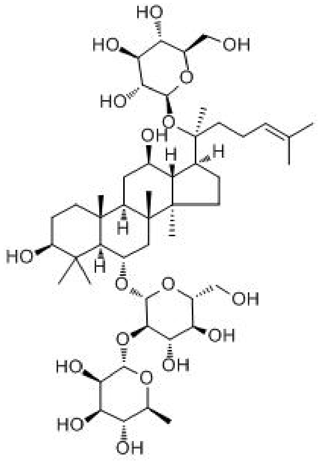	ginsenoside Re	Glc(2→1) Rha	Glc	182

8	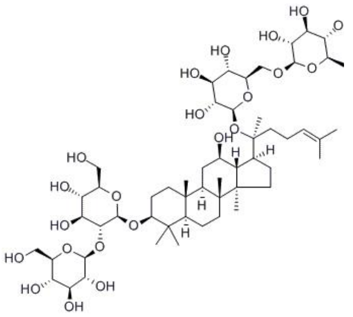	ginsenoside Rb1	Glc(2→1) Glc	Glc(6→1) Glc	366
12	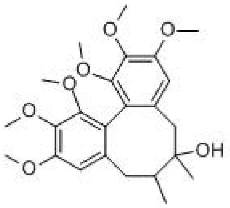	schisandrin			19.13

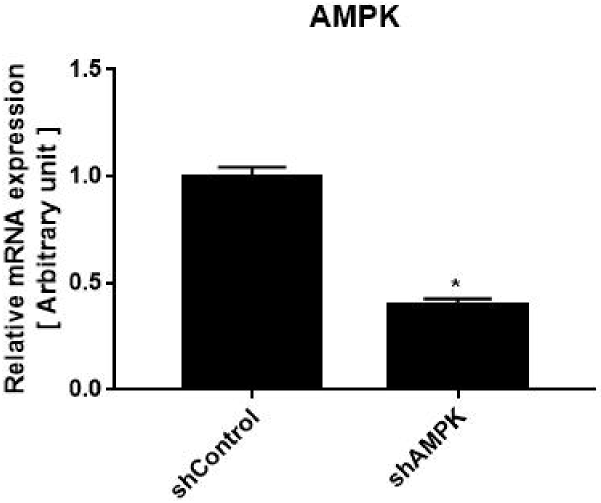

Supplementary Figure 4. qRT-PCR quantification of AMPK mRNA levels (normalized to GADPH levels) in rat neonatal cardiomyocytes with recombinant lentivirus AMPK-shRNA, or control-shRNA (*, *P* <0.05, *n*=3).
